# Automated brightfield morphometry of 3D organoid populations by OrganoSeg

**DOI:** 10.1038/s41598-017-18815-8

**Published:** 2018-03-28

**Authors:** Michael A. Borten, Sameer S. Bajikar, Nobuo Sasaki, Hans Clevers, Kevin A. Janes

**Affiliations:** 1 Department of Biomedical Engineering, University of Virginia, Charlottesville, Virginia USA; 2 Hubrecht Institute for Developmental Biology and Stem Cell Research, 3584 CT Utrecht, The Netherlands; 3 Department of Gastroenterology, Keio University School of Medicine, Tokyo, Japan

## Abstract

Spheroid and organoid cultures are powerful *in vitro* models for biology, but size and shape diversity within the culture is largely ignored. To streamline morphometric profiling, we developed OrganoSeg, an open-source software that integrates segmentation, filtering, and analysis for archived brightfield images of 3D culture. OrganoSeg is more accurate and flexible than existing platforms, and we illustrate its potential by stratifying 5167 breast-cancer spheroid and 5743 colon and colorectal-cancer organoid morphologies. Organoid transcripts grouped by morphometric signature heterogeneity were enriched for biological processes not prominent in the original RNA sequencing data. OrganoSeg enables complete, objective quantification of brightfield phenotypes, which may give insight into the molecular and multicellular mechanisms of organoid regulation.

## Introduction

3D spheroid and organoid cultures have dramatically expanded in scope and utility^1,2^. These cultures often exhibit variable size and morphologic phenotypes, but rarely are such heterogeneities analyzed systematically^3–7^, even though shape reflects and impacts single-cell regulation^8–11^. For large-scale morphometric profiling^8,12^, digital segmentation and analysis are essential^13,14^, but new considerations arise with 3D multicellular cultures^15^. Organoids grown within or atop extracellular matrix hydrogels do not reside in the same focal plane, causing nonuniform blur in transmitted-light images, even when collected with low-magnification, low-NA objectives providing a large depth of field. Optical sectioning by confocal microscopy is generally impractical because of the nonconventional working distances involved. The 3D hydrogel medium adds spherical aberration and brightfield noise, which corrupt the phase- and differential-interference contrast images normally used to document organoid growth longitudinally. Therefore, despite opportunities for unbiased assessments of organoid diversity, low-magnification brightfield images are more likely to be archived, scored by hand, or cropped tightly as “representative displays”.

## Results

**Figure 1:**
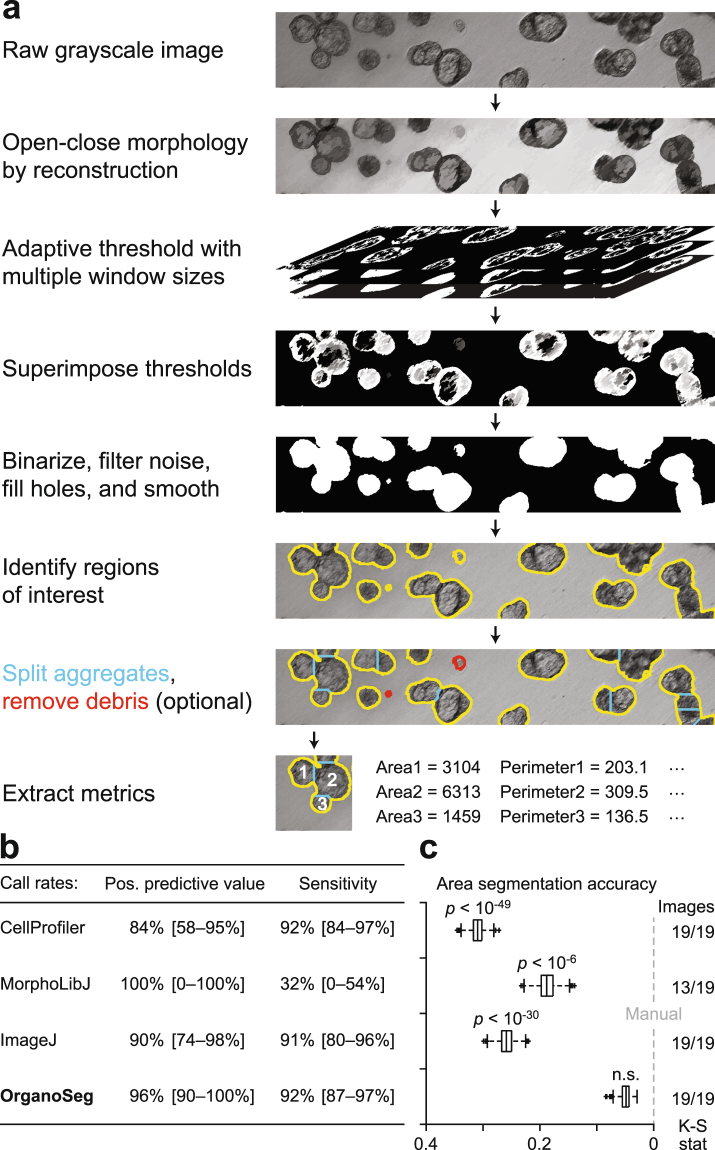
The OrganoSeg pipeline segments brightfield 3D-culture images more accurately than existing methods. (**a**) Key steps in the image-segmentation pipeline applied to a representative brightfield image. Users can opt to split aggregates (blue) or remove debris (red) in the software. (**b**,**c**) Comparison of OrganoSeg with competing alternatives^13–15^ according to spheroid call rates in (**b)** and Kolmogorov-Smirnov statistics (K-S stat) of segmented area distributions in (**c**). The number of images segmentable by each algorithm is shown to the right of **c**. Images were segmented with following OrganoSeg parameters: Otsu threshold = 1, Max-window size = 250 pixels, Size-exclusion threshold = 10 pixels. Data in (**b**) are shown as the median call rate with 95% confidence intervals in brackets from *n* = 19 images of MCF10A-5E spheroids. Data in (**c**) are shown as median boxplots of K-S stat from 1000 bootstrap replicates of *n* = 861 total spheroids, with significant differences from manual segmentation (gray) assessed by K-S test.

We addressed these challenges in the standalone software package, OrganoSeg, which provides an intuitive, graphical user interface for quantifying transmitted-light images of 3D spheroid and organoid cultures (Supplementary File S1). Starting with a grayscale or grayscale-converted image (tif,jpg,png), OrganoSeg applies morphological open-close operations that smooth bulk objects while retaining sharp spheroid boundaries (Fig. 1a). To binarize the smoothed image, we developed a variant of local adaptive thresholding^16^ that tests a range of local window sizes, creating a stack of binary images whose border-cleared union defines the final image mask. Within each window, a modulated Otsu’s method is used for binarization according to a user-defined, background-foreground sensitivity parameter reflecting image contrast. By compiling a range of window sizes, the OrganoSeg implementation reduces the impact of any given window on the final adaptive threshold. After bulk segmentation, users can define a minimum size threshold to exclude debris or organoids with poor growth, and a sphere-splitting option is provided to partition overlapping structures in densely seeded 3D spheroid cultures. From each segmented object, the software extracts up to 23 standard measures of morphometry and texture for downstream analysis (Supplementary Table S1). The OrganoSeg pipeline is compatible with brightfield, phase-contrast, and differential-interference contrast images and readily segmented images or movies of 3D-cultured breast, mammary fragments, pancreas, and colorectal cancer from independent laboratories (Supplementary Fig. S1 and Supplementary Video S1).

For automated morphometry to be useful and reliable, a segmentation pipeline must distinguish true objects from artifacts and accurately demarcate spheroid-organoid borders. We quantified spheroid call rates and extracted area distributions for a pooled collection of 861 MCF10A-5E breast-epithelial spheroids^17^ (Supplementary Fig. S2). Spheroid images were aggregated across four different laboratories to capture batch variation stemming from different hydrogel lots and brightfield imaging setups. The images were manually traced as a standard reference with which to compare OrganoSeg against CellProfiler^13^, MorphoLibJ^15^, and ImageJ^14^. The existing platforms each implemented the segmentation pipeline to the extent possible, but all lacked the multi-window local adaptive thresholding of OrganoSeg (Fig. 1a). Compared to the manual reference, automated spheroid call rates were highest for ImageJ and OrganoSeg (Fig. 1b). CellProfiler showed the lowest positive predictive value in this setting, because debris and other imaging irregularities were incorrectly segmented as spheroids (Fig. 1b and Supplementary Fig. S3). Conversely, MorphLibJ exhibited low sensitivity, as many spheroids and some entire images were left unsegmented by the algorithm. The overall fidelity of automated segmentation was assessed by using spheroid area to gauge segmentation accuracy and K-S statistics to compare area distributions nonparametrically^18^. OrganoSeg significantly outperformed competing alternatives including ImageJ, which yielded area distributions 5.4-fold more different than the manual reference relative to OrganoSeg (Fig. 1c). The side-by-side comparison with existing platforms emphasized the need for a dedicated approach to 3D spheroid and organoid cultures as provided by OrganoSeg.

**Figure 2:**
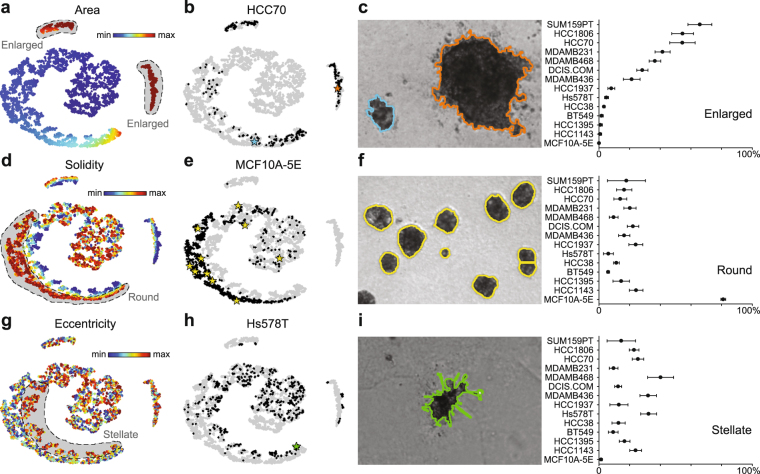
Data-driven classification and quantification of spheroid states by OrganoSeg. (**a–c**) Spheroid polydispersity is associated with an enlarged state in a fraction of triple-negative breast cancer lines. (**d–f**) A discrete round state is specifically enriched in nontransformed MCF10A-5E 3D cultures. (**g–i**) Reproducible differences in stellate invasion among triple-negative breast cancer lines. tSNE plots in (**a**,**d**,**g**) are pseudocolored by the weight of each image metric in the projection, and gates for the indicated spheroid states (gray) are dashed. Projections of the indicated cell lines in (**b**,**e**,**h**) are shown alongside specific brightfield examples (stars). Images were segmented with following OrganoSeg parameter ranges: Otsu threshold = 0.183–1, Max-window size = 100–370 pixels, Size-exclusion threshold = 148–1130 pixels. Data in (**c**,**f**,**i**) are shown as the mean ± s.d. of *n* = 4 cultures together containing 102–1069 spheroids.

We next leveraged OrganoSeg to analyze a panel of brightfield images for 14 triple-negative breast cancer or epithelial lines that are widely cultured as 3D spheroids^7,17,19^ (Supplementary Fig. S4). The resulting dataset of 5167 spheroids x 23 image metrics was visualized by *t*-distributed stochastic neighbor embedding (tSNE) to identify discriminating image features and cell-line projections^20^. Among the three islands on the tSNE map, two were heavily represented by cell area (Fig. 2a). Gating these islands provided an unbiased way to score enlarged structures in polydisperse cultures, such as HCC70 spheroids (Fig. 2b). We scored the 14 lines for enlargement and observed a pronounced increase in polydispersity for half of them (Fig. 2c). The split did not coincide with triple-negative subtype or p53 status (Supplementary Table S2), indicating that morphometric phenotyping adds information beyond molecular classifiers. Besides area, we noted a third tSNE island that was clearly segregated by eccentricity and solidity metrics, which allowed us to define further gates for round and stellate clones within the 3D cultures (Fig. 2d–i). As expected^17^, nontransformed MCF10A-5E cultures were the most round and least stellate (Fig. 2e,f,i). More surprising was the consistent proportion of 10–30% round spheroids in all of the triple-negative breast cancer lines (Fig. 2f), many of which exhibited considerable stellate invasion (Fig. 2i). The automated morphometry data reinforce the notion that triple-negative breast cancer cells undergo various reversible transitions between regulatory states^21,22^. These results illustrate how combining OrganoSeg with dimensionality reduction enables objective gating schemes for classifying morphometric phenotypes.

**Figure 3:**
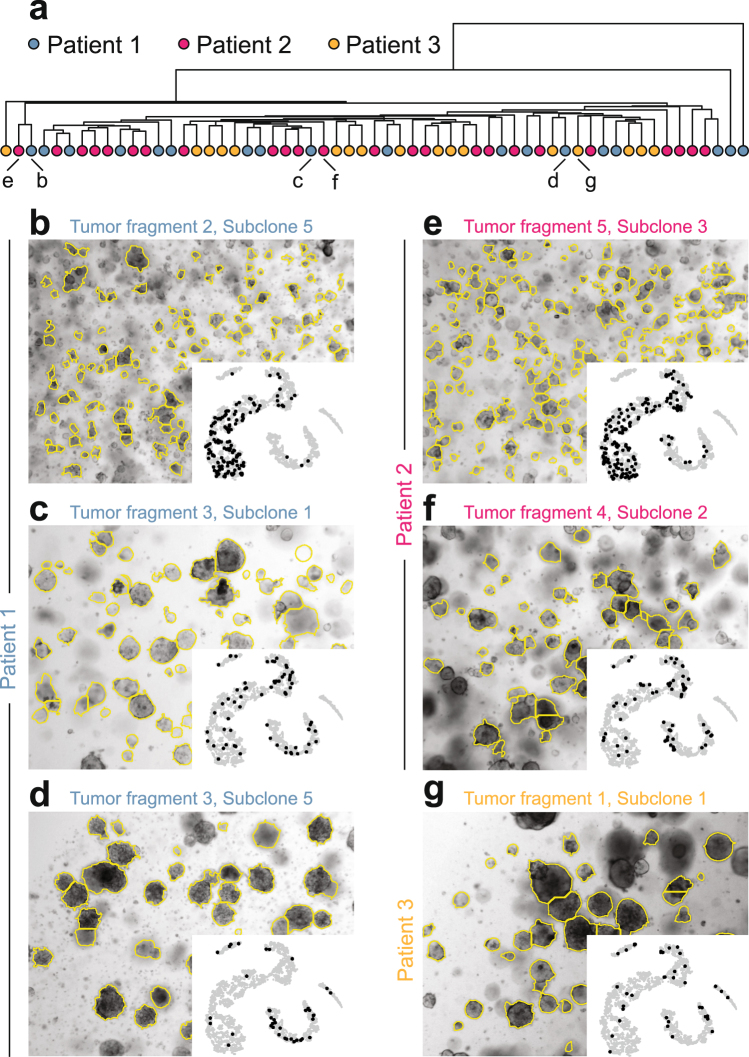
OrganoSeg identifies quantitative heterogeneity and commonality among colorectal cancer organoids. (**a**) Hierarchical clustering of tSNE projections (Supplementary Fig. S5) binned for 10–434 organoids in 64 subclones across three patients^23^. The clustering position of specific image subpanels is indicated. (**b–d**) Subclones within patients exhibit substantially different size distributions and optical-density features. (**e–g**) Examples of subclones in other patients with binned tSNE projections similar to those in (**b**) to (**d)**. The insets of (**b)** to (**g)** show the projection of the corresponding subclone on the tSNE plot of all 4940 tumor organoids in the dataset. Images were segmented with following OrganoSeg parameter ranges: Otsu threshold = 0.147–1, Max-window size = 30–260 pixels, Size-exclusion threshold = 50–2000 pixels.

To test the general utility of OrganoSeg for diverse applications, we segmented 5743 organoids subcloned from normal tissue (*n* = 803) and tumor fragments (*n* = 4940) of three colorectal cancer patients^23^. Organoid preparations were embedded in basement membrane extract, giving rise to many defocused organoids within the widefield image plane. Nevertheless, OrganoSeg proficiently segmented the in-focus or near-focused organoids for metric extraction and tSNE projection (Supplementary Fig. S5 and Supplementary Fig. S6). When the unsupervised tumor tSNE projections were clustered for each patient, tumor fragment, and subclone, we noted a remarkable heterogeneity among the preparations (Fig. 3a). Within any patient, there were substantial variations in the distribution of organoid size and optical density among different tumor fragments and even among subclones within a fragment (Fig. 3b–d). Yet, we often identified highly similar tumor organoid preparations from other patients based on the tSNE projection of OrganoSeg data (Fig. 3e–g), raising the possibility that such states recur across colorectal cancers in different proportions. Achieving similar brightfield comparisons would be laborious by manual segmentation and impossible by visual inspection.

**Figure 4:**
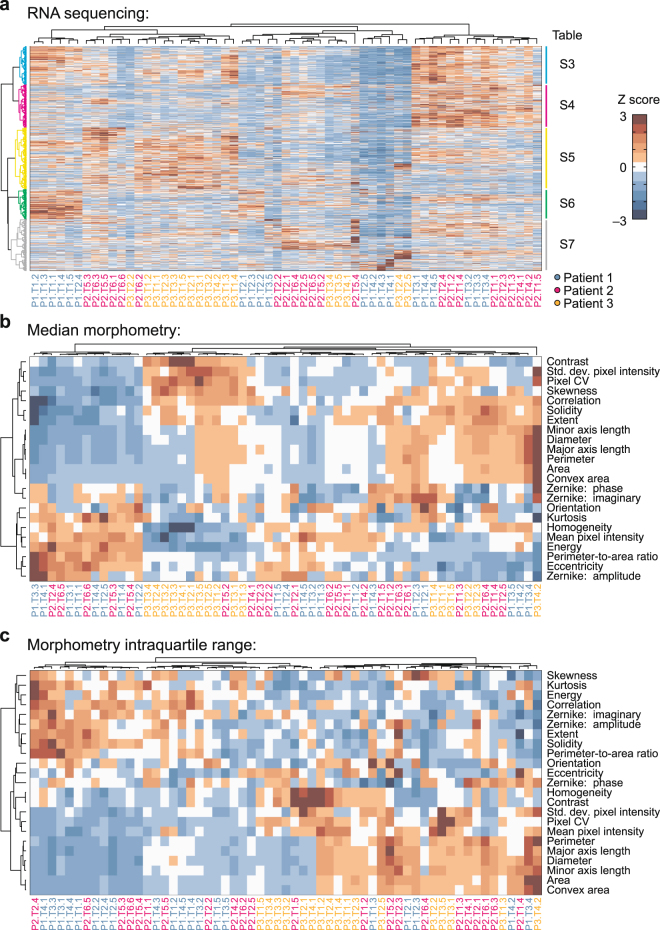
Morphometric profiling fuses colorectal cancer organoids that patient-segregate by RNA sequencing. (**a**) Hierarchical clustering of RNA sequencing data^23^ for the indicated patient (P), tumor fragment (T), and subclone. Enriched Gene Ontology biological processes for the indicated gene clusters are included in Supplementary Tables S3–S7. (**b**,**c**) Hierarchical clustering of morphometric profiles based on median OrganoSeg metrics in (**b**) and interquartile range in (**c**).

We evaluated whether morphometric heterogeneity associates with biomolecular changes by analyzing bulk RNA sequencing data from the colorectal cancer organoids^23^. Transcriptomic profiles of the different organoid preparations clustered strongly by patient (Fig. 4a), and the five largest gene clusters were selected for Gene Ontology (GO) analysis^24,25^. We observed strong and significant enrichments in the following biological processes: mitosis (cyan), sterol and alcohol biosynthesis (magenta), mitochondrial translation and electron-transport-chain assembly (yellow), ribosome biogenesis (green), and type I interferon signaling (gray) (Fig. 4a and Supplementary Tables S3–7). In these organoids, variability in mRNA expression is dominated by patient-to-patient differences in proliferation, metabolism, growth, and signaling.

To pursue a similar clustering by morphometric signature, we calculated the median and interquartile range of each metric across all organoids in a preparation. Consistent with the tSNE mapping of individual organoids (Supplementary Fig. S6), there was considerable patient overlap when preparations were grouped by morphometry (Fig. 4b,c). One patient (P3) was rather different than the other two. Nonetheless, compared to RNA sequencing, we observed far fewer instances of segregation, defined as three contiguously co-clustering preparations from the same patient (*p* < 0.05 by hypergeometric test). These observations suggested that OrganoSeg morphometry profiles were more robust to inter-individual differences and may identify stronger associations with cell phenotype when used together with transcriptomics.

**Figure 5:**
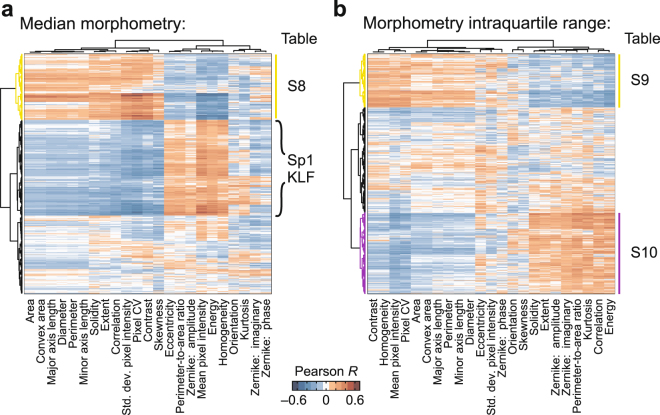
mRNA-morphometry associations suggest new hypotheses for organoid regulation. (**a,b**) Hierarchical clustering of Pearson correlations between transcript abundance and median OrganoSeg metrics in (**a)** and interquartile range in (**b**). Enriched Gene Ontology biological processes for the indicated gene clusters are included in Supplementary Tables S8–S10. Braces in (**a**) indicate gene-set enrichment for Sp1/Krüppel-like factor (KLF) binding sites in the cluster.

We explored this possibility by correlating each transcript with the associated image-metric statistics for all organoid preparations. Few individual gene-metric correlations were significant after correction for false discovery, but by clustering all measures, we reasoned that broader transcriptional programs could be identified. Applying this approach to the median OrganoSeg data revealed a group of genes associated with organoid size and pixel variation (Fig. 5a, yellow). The cluster was enriched for many of the same mitochondrial translation and electron-transport-chain assembly processes as identified by RNA sequencing (Fig. 4a, yellow and Supplementary Table S8). Another cluster was anticorrelated with the first but did not strongly enrich for any GO biological processes, suggesting that these genes may instead act as readouts of an upstream transcriptional program. Using MSigDB to perform gene set enrichment analysis^26,27^, we found a highly significant enrichment for binding sites in the Sp1/Krüppel-like factor family^28^ (*p* < 10^−144^ by Bonferroni-corrected hypergeometric test) and for genes downregulated in *Klf1*^*−/−*^ cells (*p* < 10^−57^). The gene group contains 11 Krüppel-like factors and SP1, which together could give rise to organoids that are smaller and more optically uniform.

For interquartile range, we again found a mitochondrial signature associated with variation in many of the same size and pixel-heterogeneity measures that previously clustered by median (Fig. 5a,b, yellow and Supplementary Table S9). Additionally, a new cluster arose from genes that correlated with large spreads in roundness, Zernike moments, and pixel distribution (Fig. 5b, purple). This cluster of genes was enriched for targets of p53 signaling and effectors of the G1 DNA damage checkpoint (Supplementary Table S10). For the co-associating metrics, we noted that the largest interquartile ranges were grouped in the leftmost two clusters of the original OrganoSeg data (Fig. 4c). These two clusters were significantly enriched in organoid preparations from patient P1 (*p* < 10^−4^ by hypergeometric test), the one patient retaining two wildtype *TP53* alleles^23^. We interpret the enriched p53 signature as evidence of a variably engaged proliferation-arrest program that impacts organoid shape and pixel diversity.

## Discussion

OrganoSeg streamlines the large-*n* morphometric profiling that should accompany studies involving 3D cultures of spheroids and organoids. By avoiding a requirement for confocal or high-contrast fluorescence micrographs, the pipeline converts previously archived images into rich, quantifiable datasets. Organoid responses feed back on the culture over many weeks, unearthing phenotypes that self-reinforce at the single-cell level. Using OrganoSeg, heterogeneity within 3D cultures can be approached systematically as a biological variable subject to modulation by paracrine factors and cellular genotype. Very recently, for example, we applied OrganoSeg to quantify the impact of a diffusible factor on 3D organization and uniformity of triple-negative breast cancer spheroids^29^.

Large-scale analysis of organoid shapes may reveal a discrete set of reference morphologies whose fractional occupancy provides insight into the cellular environment or genetic perturbation of the culture^12^. We gleaned hints of such relationships in colorectal cancer preparations by combining OrganoSeg with RNA sequencing to highlight biology missed by RNA sequencing alone. Conceivably, the same benefits would apply to metabolomic (Supplementary Tables S5, S8 and S9) or proteomic datasets paired with OrganoSeg. Longitudinal imaging of 3D-culture trajectories (Supplementary Fig. S1b) would provide even richer morphometric signatures to connect with terminal –omic data types. The ease of acquisition—and, now, segmentation—justifies incorporating brightfield analysis into standard 3D phenotyping procedures involving spheroid-organoid culture. OrganoSeg is available as a standalone executable for open distribution and also as a MATLAB script for future extensions of its capabilities (Supplementary File S1).

## Methods

### OrganoSeg

OrganoSeg was constructed in MATLAB using the Image Processing Toolbox. The algorithm is comprised of three main steps: 1) smoothing, 2) adaptive thresholding, and 3) post-segmentation processing. For smoothing, raw grayscale images were sequentially sharpened with imsharpen (radius = 1, amount = 0.8, threshold = 0), median filtered with medfilt2 (neighborhood = [3,3]), opened by reconstruction with imerode (structuring element = 5-pixel radius disk) followed by imreconstruct (marker = eroded image, mask = non-eroded image), and closed by reconstruction with imdilate (structuring element = 5-pixel radius disk) followed by imreconstruct (marker = dilated image, mask = non-dilated image) to arrive at the final smoothed image.

Adaptive thresholding was based off of the adaptivethreshold function (MATLAB File Exchange #8647). Smoothed images were averaged filtered with imfilter (boundary = replicate) using fspecial (filter type = average) and a range of window sizes from 20 pixels to a user-defined Max-window size (default = 250 pixels) in 10-pixel increments. Next, the original image was subtracted from the average-filtered image, and the resulting difference was binarized by global Otsu gray threshold with imbinarize (threshold = defined by the graythresh function multiplied by a user-defined Otsu threshold parameter) for each window size. The stack of binarized images across window sizes was combined by addition to yield a binary image with more detail than a binary image formed from a single window size.

For post-segmentation processing, the binary image was size thresholded with bwareaopen (maximum area = user-defined Size-exclusion threshold parameter), closed with imclose (structuring element = 3 × 3 square), hole filled with imfill (parameter = holes), and border cleared with imclearborder. Metrics were extracted from the final binary image by concatenating the outputs of regionprops (parameter = all), graycomatrix (Symmetric = true), graycoprops, and Zernicke_main (MATLAB File Exchange #38900) (Supplementary Table S1).

For segmenting DIC images, adaptive thresholding (step 2) was performed on both the normal smoothed image, and an inverted smoothed image created with imcomplement. The resulting binarized images were combined by addition to yield a final binary image. For DIC images, a fixed window size must be selected (default = 20 pixels).

Conjoined objects have the option of being split by using the watershed algorithm. Before border clearing, the distance transform of the binary image was calculated with bwdist, smoothed with imgaussfilt (sigma = 5), and non-object pixels were set to −∞ before applying the watershed algorithm. The split binary image was size thresholded again with bwareaopen (maximum area = user-defined parameter) and finally border cleared with imclearborder.

OrganoSeg source code, Windows-compatible executable, Mac-compatible executable, and instructional materials are available in Supplementary File S1.

### Alternative segmentation pipelines

We sought alternative pipelines that could mimic the smoothing, adaptive thresholding, and post-segmentation steps of OrganoSeg to the extent possible by the software. Three alternative segmentation approaches were tested: CellProfiler^13^, MorphoLibJ^15^, and ImageJ^14^. For CellProfiler, smoothing was achieved by opening with Morph/open (structuring element = 5 pixel diameter disk) and closing with Morph/close (structuring element = 5 pixel diameter disk). Adaptive thresholding of the smoothed image was performed by applying a Gaussian filter with CorrectIlluminationCalculate/GaussianFilter (size = 50 pixels), dividing the original image by the Gaussian-filtered image with CorrectIlluminationApply (parameter = divide), and segmenting the divided image by Otsu’s method with IdentifyPrimaryObjects/Global/Otsu (parameters = two-class thresholding, weighted variance minimized). Post-segmentation processing involved hole filling with IdentifyPrimaryObjects/Fill holes in identified objects and excluding small objects with IdentifyPrimaryObjects (1 pixel < diameter of objects < 50000 pixels). Small objects were excluded by extracting areas with MeasureObjectSizeShape and eliminating extracted objects smaller than 10 pixels after export from CellProfiler. The CellProfiler script is available in Supplementary File S2.

For the MorphoLibJ plugin, scripting is not available, so implementation was limited to the settings available through the graphical user interface, which were manually optimized to find the best batch segmentation. Images were analyzed with Object Image (gradient radius = 1, watershed tolerance = 35). The segmentation was exported as Watershed Lines, and objects were hole filled with Fill Holes before identification with Analyze Particles (size ≥ 10, exclude add).

For ImageJ, smoothing was achieved by sharpening the raw image with Sharpen, median filtering with Median (radius = 1), opening by reconstruction with Maximum (radius = 5) followed by Morphological Reconstruction (marker = eroded image, mask = non-eroded image), and closing by reconstruction with Minimum (radius = 5) followed by Morphological Reconstruction (marker = dilated image, mask = non-dilated image). Adaptive thresholding of the smoothed image was performed with the adaptiveThr plugin (parameter = weighted-mean, window size = 250) (https://sites.google.com/site/qingzongtseng/adaptivethreshold). Post-segmentation processing involved hole filling with Fill Holes and size thresholding plus border removal with Analyze Particles (size ≥ 10, parameter = exclude add). Area metric was extracted with ROI Manager (measure). The ImageJ script is available in Supplementary File S2.

### Comparison to manual segmentation

Images acquired from four independent laboratories were visually identified and manually segmented in ImageJ, ignoring objects touching the image border. Segmented objects were captured as regions of interest, and the area of each object was extracted with ROI Manager. To compare positive predictive value and sensitivity, the manually segmented objects were extracted as masks and compared with the masks generated by OrganoSeg and the alternative segmentation pipelines. Object identifications were scored positive if any pixels of an automatically segmented object contained pixels in a manually segmented object from that image. Negative identifications were assigned to manually segmented objects that did not overlap with any automatically segmented objects from that image. False-positive identifications were assigned to automatically segmented objects that did not overlap with any manually segmented objects from that image. Positive predictive value was calculated as positives divided by positives + false positives. Sensitivity was calculated as positives divided by the total number of manually segmented objects.

To compare segmentation fidelity, object areas were normalized for each image to the overall image area to account for the different scales of the four independent image sources. The cumulative distribution function of all normalized objects (as defined by each pipeline) was compared by Kolmogorov-Smirnov (K-S) statistics with confidence intervals estimated from 1000 bootstrapped replicates of the segmented objects.

### Cell lines

All breast cancer cell lines, except for MCF10A-5E, MCF10DCIS.COM, and SUM159PT, were obtained from ATCC and cultured as recommended. The MCF10A-5E subclone of the MCF10A parental line was previously described and cultured as before^17^. The MCF10ADCIS.COM cell line was acquired from Wayne State University and cultured as previously described^22^. The SUM159PT cell line was acquired from Asterand Bioscience and cultured as recommended, except growth media formulation was supplemented with 100 units/mL Penicillin and 100 µg/ml Streptomycin (Gibco #15140-122).

### Triple-negative breast cancer spheroid culture

3D overlay cultures were performed on top of Matrigel (BD Biosciences) as described previously for MCF-10A cells^30^ with seeding conditions and culture media optimized for each cell line (Supplementary Table S11). 3D culture medium was replaced every four days as previously described^30^. Brightfield images were acquired every 3–5 days by using a 4× plan achromat objective on an Olympus CKX41 microscope mounted with a qColor3 camera. Images were white balanced (after preliminary autoexposure) and autoexposed with 2 × 2 pixel binning. Four images were acquired per chamber well, and at least three wells were imaged per condition. Each image was segmented by individually optimizing the OrganoSeg parameters manually until a suitable segmentation was achieved; specific parameter ranges are reported in each figure caption. For comparison between cell lines (Fig. 2), the time point analyzed for each cell line was selected for maximal spheroid growth but before overgrowth into adjacent spheroids: Day 12 (SUM159PT), Day 16 (HCC1806, Hs578T, MCF10ADCIS.COM, and MDA-MB-468), or Day 20 (BT-549, HCC38, HCC70, HCC1143, HCC1395, HCC1937, MCF10A-5E, MDA-MB-231, and MDA-MB-436). Raw images, MATLAB .mat files, and extracted metrics are available in Supplementary File S3.

### External spheroid and organoid images

MCF10A-5E spheroids were cultured according to standard procedures^30^ and imaged by four independent laboratories with distinct microscopy setups. Pancreatic organoids and mammary fragments were cultured and imaged as described^31,32^.

### Colorectal cancer organoid culture

Normal and tumor organoid cultures derived from three colorectal cancer patients were isolated and prepared as described^23^. Raw images, MATLAB .mat files, and extracted metrics are available in Supplementary File S3.

### tSNE data reduction and hierarchical clustering

tSNE data reduction was performed in MATLAB with the tsne function (initial dimensions = 23 image metrics, final dimensions = 2, perplexity = 30) of van der Maaten^20^ without pre-standardization. Each tSNE map was calculated at least three times to select a final mapping that was representative and most visually interpretable. Hierarchical clustering of the colorectal cancer organoid tSNE map (Fig. 3a) was performed by partitioning the map into a 15 × 15 grid of bounding boxes. The percentage of observations falling into each bounding box created a 15 × 15 = 225 bounding-box signature that was calculated for each organoid sample in the tSNE map. Hierarchical clustering was performed in MATLAB with the clustergram function (clustering = row, standardization = none, linkage = Ward).

### RNA sequencing alignment and analysis

Raw RNA sequencing data from the colorectal cancer organoids^23^ was downloaded from the European Genome-phenome Archive (EGAD00001003320) and coded by patient, tumor fragment, and subclone according to the identifier key provided by S.F. Roerink (Supplementary File S4). Downloaded CRAM files were converted to genome-aligned BAM files using SAMtools and then FASTQ files using BEDtools. The resulting FASTQ files were quasi-mapped to the human hg19 transcriptome and quantified with Salmon^33^ before normalizing as transcripts per million (TPM). Aligned and quantified TPM data are available in Supplementary File S5.

The quantified RNA sequencing data was intersected with the 59 colorectal cancer organoid preparations for which brightfield images were available. Reference ensGene transcripts with identical gene names and quantification were removed as duplicates along with mitochondrial transcripts, and the data were renormalized as TPM. The uniquely quantified mRNAs were then filtered to remove transcripts with a median TPM less than one (~0.2 copies per cell according to Ref.^34^). The intersected and filtered TPM data are available in Supplementary File S6. Hierarchical clustering of filtered data and Pearson correlations with morphometry was performed in MATLAB with the clustergram function (clustering = row and column, standardization = row, linkage = Ward).

### GO analysis and gene set enrichment analysis

Gene names from the indicated clusters of Figs. 4 and 5 were evaluated for enriched biological processes by GO analysis^24,25^ (http://geneontology.org). Processes enriched at least 2.5-fold are included in Supplementary Tables S3–S10. Gene set analysis of the indicated cluster in Fig. 5a was performed against all gene sets and signatures curated in MSigDB^26,27^ (http://software.broadinstitute.org/gsea/msigdb). The large size of the cluster (4882 genes) exceeded the 2940-gene limit for web-based calculation of enrichment. Therefore, the cluster was divided into three segments, and signatures enriched in two of three segments were assessed offline for enrichment by hypergeometric test followed by Bonferroni correction for multiple-hypothesis testing across the 17,786 signatures currently deposited in MSigDB.

### Data availability

The MATLAB code and OrganoSeg standalone executable files are available in Supplementary File S1. All raw brightfield images, MATLAB .mat files, and extracted metrics are available in Supplementary File S3. Quantified RNA-sequencing alignments are available in Supplementary Files S5 and S6. Raw RNA-sequencing data were obtained under contract with the European Genome-phenome Archive for the current study and can be obtained upon reasonable request with permission from the Wellcome Trust Sanger Institute.

## Electronic supplementary material

Supplementary Information

Files S1-S3

File S4

File S5

File S6

SI 1

SI 2

SI 3

Video S1

## References

[1] Shamir ER, Ewald AJ (2014). Three-dimensional organotypic culture: experimental models of mammalian biology and disease. Nat. Rev. Mol. Cell Biol..

[2] Clevers H (2016). Modeling Development and Disease with Organoids. Cell.

[3] Muthuswamy SK, Li D, Lelievre S, Bissell MJ, Brugge JS (2001). ErbB2, but not ErbB1, reinitiates proliferation and induces luminal repopulation in epithelial acini. Nat. Cell Biol..

[4] Freed-Pastor WA (2012). Mutant p53 disrupts mammary tissue architecture via the mevalonate pathway. Cell.

[5] Lancaster MA, Knoblich JA (2014). Generation of cerebral organoids from human pluripotent stem cells. Nat. Protoc..

[6] Rock JR (2009). Basal cells as stem cells of the mouse trachea and human airway epithelium. Proc. Natl. Acad. Sci. USA.

[7] Kenny PA (2007). The morphologies of breast cancer cell lines in three-dimensional assays correlate with their profiles of gene expression. Mol. Oncol..

[8] Bakal C, Aach J, Church G, Perrimon N (2007). Quantitative morphological signatures define local signaling networks regulating cell morphology. Science.

[9] Rangamani P (2013). Decoding information in cell shape. Cell.

[10] Chen CS, Mrksich M, Huang S, Whitesides GM, Ingber DE (1997). Geometric control of cell life and death. Science.

[11] McBeath R, Pirone DM, Nelson CM, Bhadriraju K, Chen CS (2004). Cell shape, cytoskeletal tension, and RhoA regulate stem cell lineage commitment. Dev. Cell.

[12] Yin Z (2013). A screen for morphological complexity identifies regulators of switch-like transitions between discrete cell shapes. Nat. Cell Biol..

[13] Carpenter AE (2006). CellProfiler: image analysis software for identifying and quantifying cell phenotypes. Genome Biol..

[14] Schneider CA, Rasband WS, Eliceiri KW (2012). NIH Image to ImageJ: 25 years of image analysis. Nat. Methods.

[15] Legland D, Arganda-Carreras I, Andrey P (2016). MorphoLibJ: integrated library and plugins for mathematical morphology with Image. J. Bioinformatics.

[16] Bradley D, Roth G (2007). Adaptive Thresholding using the Integral Image. Journal of Graphics Tools.

[17] Janes KA, Wang CC, Holmberg KJ, Cabral K, Brugge JS (2010). Identifying single-cell molecular programs by stochastic profiling. Nat. Methods.

[18] Perlman ZE (2004). Multidimensional drug profiling by automated microscopy. Science.

[19] Lombardo Y (2012). Nicastrin regulates breast cancer stem cell properties and tumor growth *in vitro* and *in vivo*. Proc. Natl. Acad. Sci. USA.

[20] van der Maaten L, Hinton G (2008). Visualizing Data using t-SNE. J. Mach. Learn. Res..

[21] Gupta PB (2011). Stochastic state transitions give rise to phenotypic equilibrium in populations of cancer cells. Cell.

[22] Wang CC, Bajikar SS, Jamal L, Atkins KA, Janes KA (2014). A time- and matrix-dependent TGFBR3-JUND-KRT5 regulatory circuit in single breast epithelial cells and basal-like premalignancies. Nat. Cell Biol..

[23] Roerink, S. F. *et al*. Intra-tumour diversification in colorectal cancer at the single cell level. *Nature*, (In Press, 2018). 10.1038/s41586-018-0024-3 29643510

[24] Ashburner M (2000). Gene ontology: tool for the unification of biology. The Gene Ontology Consortium. Nat. Genet..

[25] The Gene Ontology, C (2017). Expansion of the Gene Ontology knowledgebase and resources. Nucleic Acids Res..

[26] Subramanian A (2005). Gene set enrichment analysis: a knowledge-based approach for interpreting genome-wide expression profiles. Proc. Natl. Acad. Sci. USA.

[27] Liberzon A (2015). The Molecular Signatures Database (MSigDB) hallmark gene set collection. Cell Syst.

[28] Kaczynski J, Cook T, Urrutia R (2003). Sp1- and Kruppel-like transcription factors. Genome Biol..

[29] Bajikar SS (2017). Tumor-Suppressor Inactivation of GDF11 Occurs by Precursor Sequestration in Triple-Negative Breast Cancer. Dev. Cell.

[30] Debnath J, Muthuswamy SK, Brugge JS (2003). Morphogenesis and oncogenesis of MCF-10A mammary epithelial acini grown in three-dimensional basement membrane cultures. Methods.

[31] Huang L (2015). Ductal pancreatic cancer modeling and drug screening using human pluripotent stem cell- and patient-derived tumor organoids. Nat. Med..

[32] Nguyen-Ngoc KV (2015). 3D culture assays of murine mammary branching morphogenesis and epithelial invasion. Methods Mol. Biol..

[33] Patro R, Duggal G, Love MI, Irizarry RA, Kingsford C (2017). Salmon provides fast and bias-aware quantification of transcript expression. Nat. Methods.

[34] Marinov GK (2014). From single-cell to cell-pool transcriptomes: stochasticity in gene expression and RNA splicing. Genome Res..

