# Supplementary material for: Automated brightfield morphometry of 3D organoid populations by OrganoSeg
Source: Sci Rep. 2018 Mar 28;8:5319. doi: 10.1038/s41598-017-18815-8 (PMC5871765; doi:10.1038/s41598-017-18815-8)
Supplement: Supplementary file 1 — Supplementary Information [file 41598_2017_18815_MOESM1_ESM.pdf]

SUPPLEMENTARY INFORMATION

Automated brightfield morphometry of 3D organoid populations by OrganoSeg

Michael A. Borten[1,4], Sameer S. Bajikar[1,4], Nobuo Sasaki[2,3], Hans Clevers[2], Kevin A. Janes[1]

[1]Department of Biomedical Engineering, University of Virginia, Charlottesville, Virginia, USA.
[2]Hubrecht Institute for Developmental Biology and Stem Cell Research, 3584 CT, Utrecht, The Netherlands
[3]Department of Gastroenterology, Keio University School of Medicine, Tokyo, Japan
[4]These authors contributed equally to this work.

Correspondence should be addressed to K.A.J. (kjanes@virginia.edu)

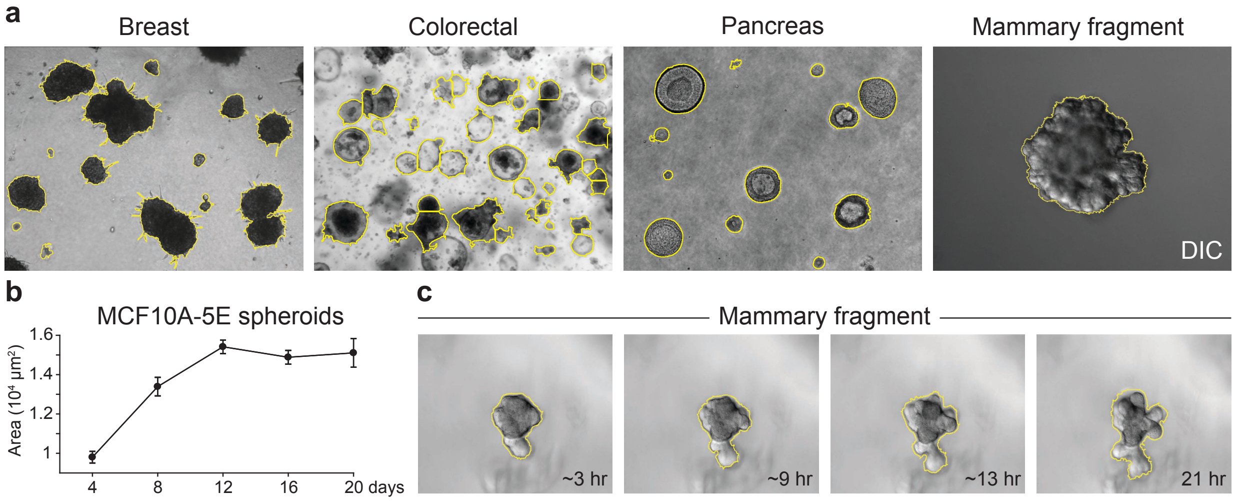

Supplementary Figure 1 | Application of OrganoSeg to different 3D culture formats and settings. (**a**) OrganoSeg accurately segments breast-cancer spheroids[1], colorectal cancer organoids (S.F. Roerink, N.S., and H.C., in revision), pancreatic organoids[2], and DIC images of mouse mammary fragments[3]. Images were segmented with following OrganoSeg parameters: Otsu threshold = 0.62–0.701 and 1 (DIC), Max-window size = 160–250 pixels and 20 pixels (DIC), Size-exclusion threshold = 100–425 pixels and 686 pixels (DIC). (**b**) Noninvasive time course of breast-epithelial spheroid growth quantified by OrganoSeg using brightfield images of MCF10A-5E cultures collected every four days. Images were segmented with following OrganoSeg parameters: Otsu threshold = 0.420–0.711, Max-window size = 100–210 pixels, Size-exclusion threshold = 127–170 pixels. (**c**) Time-lapse imaging of mammary branching with paired per-frame segmentation (**Supplementary Video 1**). Images were segmented with following OrganoSeg parameters: Otsu threshold = 0.746–0.944, Max-window size = 20 pixels, Size-exclusion threshold = 1209–2626 pixels.

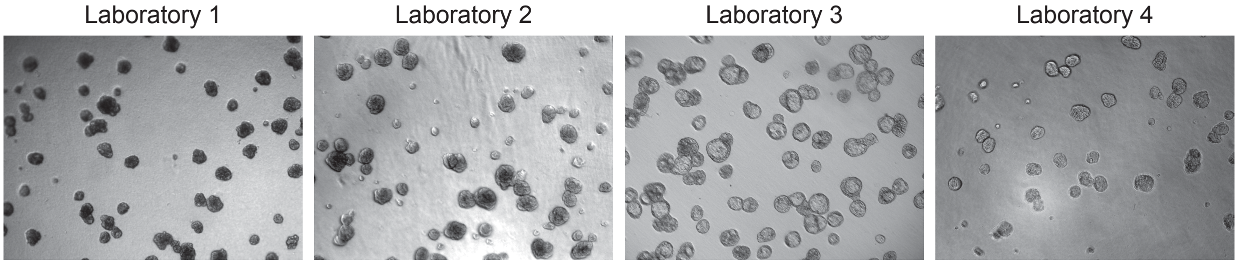

Supplementary Figure 2 | Representative images of MCF10A-5E spheroids illustrating variations in image quality and inhomogeneity. Data were obtained from four independent laboratories.

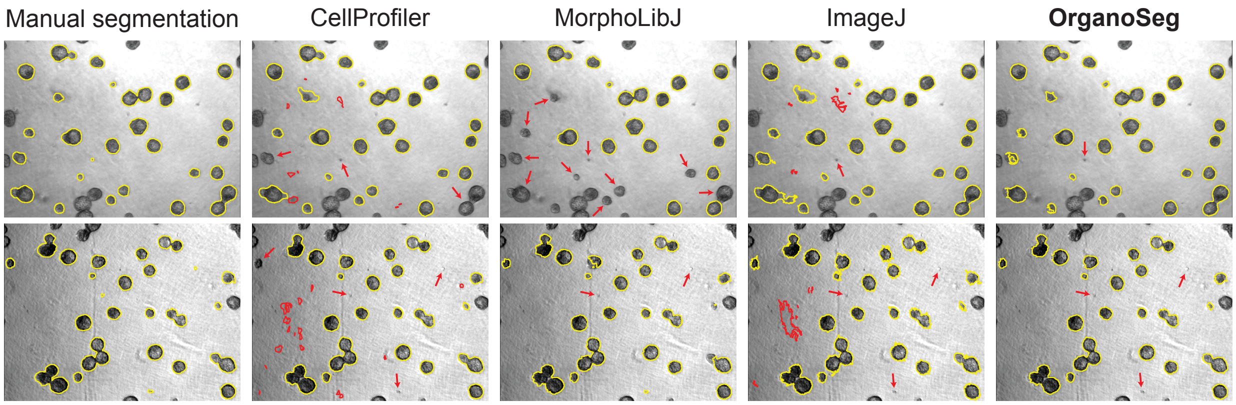

Supplementary Figure 3 | OrganoSeg outperforms CellProfiler, MorphoLibJ, and ImageJ with accuracy that is comparable to manually segmented images. Representative MCF10A-5E brightfield images are shown with falsely segmented debris (red outlines) and missed spheroids (red arrows) highlighted. Images were segmented with following OrganoSeg parameters: Otsu threshold = 1, Max-window size = 250 pixels, Size-exclusion threshold = 10 pixels.

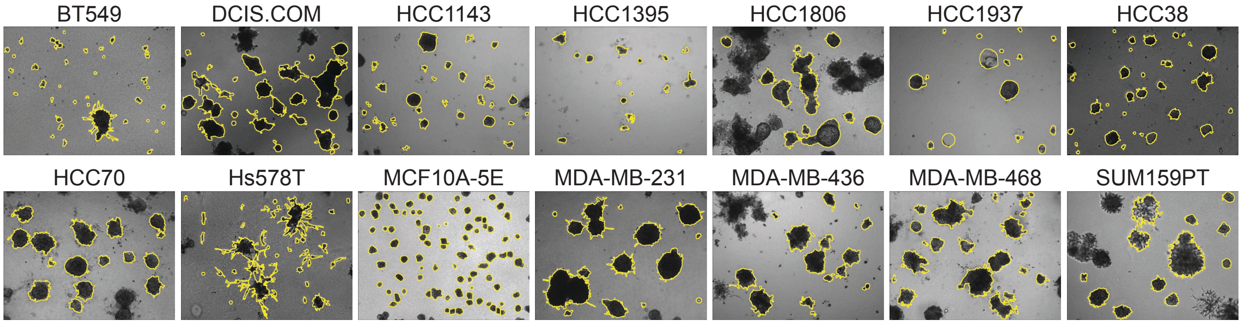

Supplementary Figure 4 | Representative OrganoSeg results for the 14 triple-negative breast lines analyzed in **Fig. 2**. Spheroids contacting the image border are cleared for the final segmentation. Images were segmented with following OrganoSeg parameters: Otsu threshold = 0.342–1, Max-window size = 100–370 pixels, Size-exclusion threshold = 148–978 pixels.

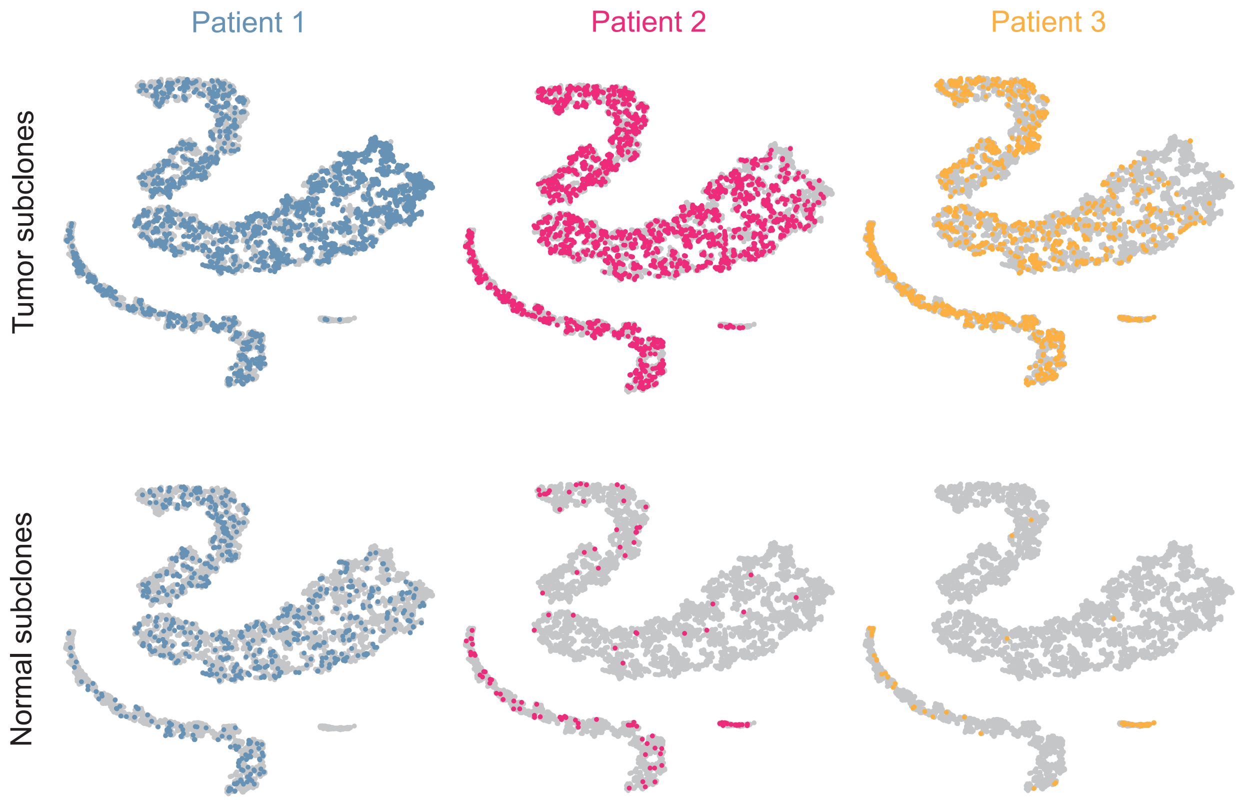

Supplementary Figure 5 | Normal and colorectal cancer organoids segregate for some patients in morphometry space. The lower two islands are enriched for organoid size metrics and segregate the normal subclones for Patients 2 and 3.

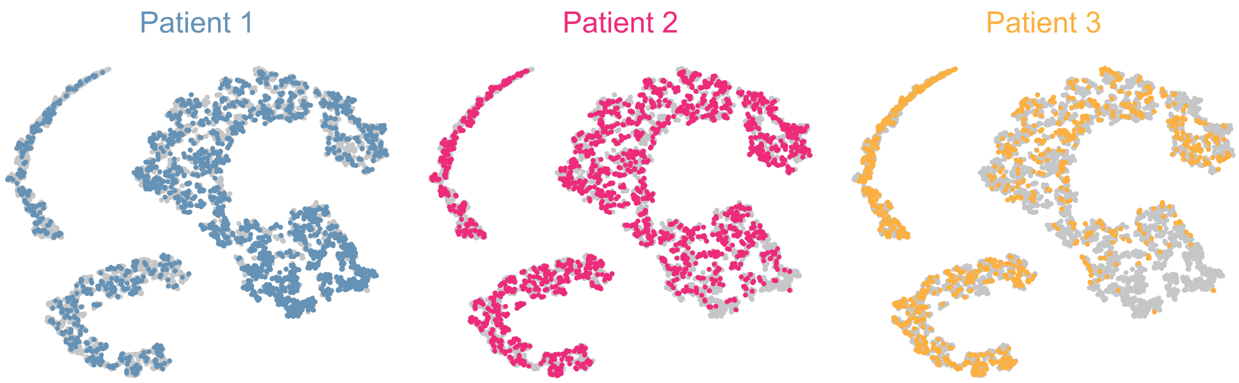

Supplementary Figure 6 | Patient-specific colorectal cancer organoids are comparably distributed in morphometry space. Normal subclones were omitted from the tSNE map.

Supplementary Table 1 | List of image metrics extracted by OrganoSeg.

Metric	Description	Range
Area	Number of pixels in the segmented region	≥ 1 pixels
Major axis length	Length of major axis of ellipse with same second central moments as segmented region	≥ 1 pixels
Minor axis length	Length of minor axis of ellipse with same second central moments as segmented region	≥ 1 pixels
Eccentricity	Ratio of distance between foci of ellipse and major axis length	0 (circle) to 1 (line)
Orientation	Angle between x-axis and major axis	–90° to 90°
Convex Area	Number of pixels in convex hull (smallest convex polygon that contains segmented region)	≥ 1 pixels
Diameter	Diameter of circle with same area as segmented region	≥ 1 pixels
Solidity	Area divided by convex area	0 (spindly) to 1 (solid)
Extent	Ratio of pixels in spheroid to pixels in total bounding box	0 (spindly) to 1 (solid/square)
Perimeter	Distance around boundary of segmented region	≥ 8 pixels
Zernike moment: imaginary	Imaginary component of complex Zernike moment of 4th order shape polynomial	≥ 0
Zernike moment: amplitude	Amplitude of complex Zernike moment of 4th order shape polynomial	≥ 0
Zernike moment: phase	Angle of complex Zernike moment of 4th order shape polynomial	–180° to 180°
Mean pixel intensity	Average pixel intensity in segmented region	0 to 255 (8-bit image)
St. dev. pixel intensity	Standard deviation of pixel intensity in segmented region	≥ 0 pixels
Perimeter-to-area ratio	Perimeter divided by area	≥ 0 pixels
Pixel coefficient of variation	Coefficient of variation of pixel intensity of segmented region (standard deviation/mean)	≥ 0
Skewness	Skewness of pixel intensity in segmented region	≥ 0
Kurtosis	Kurtosis of pixel intensity in segmented region	≥ 0
Contrast	Intensity contrast between pixel and neighbor (i.e., variance)	0 (constant) to max (variable)
Correlation	Correlation of pixel and neighbor	–1 (– correlated) to 1 (+ correlated)
Energy	Dominance of adjacent pixel combinations (i.e., uniformity)	0 (variable) to 1 (constant)
Homogeneity	Likelihood of adjacent pixels being equal (i.e., smoothness)	0 (rough) to 1 (smooth)

Supplementary Table 2 | Breast spheroid enlargement does not correlate with triple-negative subtype or p53 status.

Cell line	Enlarged (%)	Subtype[a]	Gene cluster	p53 status
MCF10A-5E	1.2 ± 0.7	n.d.[b]	Basal B	Wild type
HCC1395	1.7 ± 2.0	UM	Basal B	Mutated
BT-549	1.8 ± 1.0	M	Basal B	Mutated
HCC38	2.1 ± 1.8	BL1	Basal B	Mutated
HCC1143	3.1 ± 0.5	BL1	Basal A	Mutated
Hs578T	5.0 ± 0.5	MSL	Basal B	Mutated
HCC1937	8.6 ± 1.4	BL1	Basal A	Mutated
MDA-MB-436	24.8 ± 4.4	MSL	Basal B	Mutated
MCF10ADCIS.COM	29.9 ± 3.4	n.d.	n.d.	n.d.
MDA-MB-468	37.3 ± 2.5	BL1	Basal A	Mutated
MDA-MB-231	41.4 ± 3.5	MSL	Basal B	Mutated
HCC70	53.5 ± 3.6	BL2	Basal A	Mutated
HCC1806	54.8 ± 6.1	IM	n.d.	Mutated
SUM159PT	65.4 ± 12.1	n.d.	Basal B	n.d.

[a] Subtype: BL1, basal-like 1; BL2, basal-like 2; IM, immunomodulatory; M, mesenchymal; MSL, mesenchymal stem-like; UM, unclassified morphology
[b] n.d., not determined

Supplementary Table 3 | Enriched Gene Ontology (GO) terms for transcripts in the cyan cluster of **Fig. 4a**.

GO biological process	Total genes[a]	Genes in cluster Expected[b]	Genes in cluster Observed	Fold enrichment	*p*-value[c]
Protein localization to kinetochore (GO:0034501)	14	2	11	6.3	1.90E-02
Protein localization to chromosome, centromeric region (GO:0071459)	17	2	13	6.2	3.16E-03
Negative regulation of mitotic metaphase/anaphase transition (GO:0045841)	21	3	13	5.0	3.13E-02
Negative regulation of sister chromatid segregation (GO:0033046)	29	4	16	4.5	1.09E-02
Negative regulation of chromosome segregation (GO:0051985)	30	4	16	4.3	1.68E-02
Mitotic cytokinesis (GO:0000281)	42	5	22	4.2	3.03E-04
Negative regulation of mitotic nuclear division (GO:0045839)	37	5	18	3.9	1.42E-02
Regulation of mitotic metaphase/anaphase transition (GO:0030071)	46	6	22	3.9	1.41E-03
Regulation of metaphase/anaphase transition of cell cycle (GO:1902099)	47	6	22	3.8	2.01E-03
Sister chromatid cohesion (GO:0007062)	120	15	56	3.8	1.93E-12
Cytoskeleton-dependent cytokinesis (GO:0061640)	52	6	24	3.7	7.51E-04
Mitotic nuclear envelope disassembly (GO:0007077)	44	5	20	3.7	1.08E-02
Negative regulation of nuclear division (GO:0051784)	49	6	22	3.6	3.98E-03
Posttranscriptional gene silencing (GO:0016441)	52	6	23	3.6	2.85E-03
Sister chromatid segregation (GO:0000819)	188	23	83	3.6	3.77E-18
Nuclear envelope disassembly (GO:0051081)	48	6	21	3.5	1.07E-02
Membrane disassembly (GO:0030397)	48	6	21	3.5	1.07E-02
Regulation of chromosome separation (GO:1905818)	55	7	24	3.5	2.03E-03
Mitotic sister chromatid segregation (GO:0000070)	101	13	44	3.5	2.74E-08

Gene silencing by miRNA (GO:0035195)	44	5	19	3.5	4.04E-02
Regulation of mitotic sister chromatid separation (GO:0010965)	51	6	22	3.5	7.61E-03
Posttranscriptional gene silencing by RNA (GO:0035194)	51	6	22	3.5	7.61E-03
Regulation of chromosome segregation (GO:0051983)	96	12	41	3.4	2.65E-07
Nuclear envelope organization (GO:0006998)	87	11	37	3.4	2.65E-06
Cytokinesis (GO:0000910)	85	11	36	3.4	5.08E-06
Regulation of mRNA splicing, via spliceosome (GO:0048024)	80	10	33	3.3	4.81E-05
Metaphase plate congression (GO:0051310)	51	6	21	3.3	2.70E-02
Regulation of mitotic sister chromatid segregation (GO:0033047)	61	8	25	3.3	3.59E-03
Protein localization to chromosome (GO:0034502)	57	7	23	3.3	1.32E-02
Regulation of sister chromatid segregation (GO:0033045)	72	9	29	3.3	6.40E-04
Chromosome localization (GO:0050000)	73	9	29	3.2	8.49E-04
Mitotic nuclear division (GO:0140014)	136	17	53	3.1	1.17E-08
Establishment of chromosome localization (GO:0051303)	72	9	28	3.1	2.12E-03
Regulation of cellular response to heat (GO:1900034)	78	10	30	3.1	1.02E-03
Positive regulation of ubiquitin protein ligase activity (GO:1904668)	85	11	32	3.0	6.31E-04
Regulation of RNA splicing (GO:0043484)	120	15	45	3.0	1.82E-06
Nuclear chromosome segregation (GO:0098813)	259	32	97	3.0	9.63E-17
Regulation of ubiquitin-protein ligase activity involved in mitotic cell cycle (GO:0051439)	73	9	27	3.0	8.78E-03
Nucleus organization (GO:0006997)	147	18	54	3.0	6.44E-08
Microtubule cytoskeleton organization involved in mitosis (GO:1902850)	93	12	34	3.0	4.97E-04

Positive regulation of ubiquitin-protein ligase activity involved in regulation of mitotic cell cycle transition (GO:0051437)	77	10	28	2.9	7.74E-03
Negative regulation of ubiquitin-protein ligase activity involved in mitotic cell cycle (GO:0051436)	72	9	26	2.9	2.10E-02
Chromosome segregation (GO:0007059)	302	37	109	2.9	5.94E-18
Mitotic spindle organization (GO:0007052)	70	9	25	2.9	3.92E-02
Positive regulation of ubiquitin-protein transferase activity (GO:0051443)	104	13	37	2.9	2.61E-04
G2/M transition of mitotic cell cycle (GO:0000086)	136	17	48	2.9	3.30E-06
Regulation of ubiquitin protein ligase activity (GO:1904666)	94	12	33	2.8	1.89E-03
Protein sumoylation (GO:0016925)	77	10	27	2.8	2.32E-02
Cell cycle G1/S phase transition (GO:0044843)	123	15	43	2.8	3.45E-05
Negative regulation of chromosome organization (GO:2001251)	115	14	40	2.8	1.35E-04
Cell cycle G2/M phase transition (GO:0044839)	138	17	48	2.8	5.24E-06
Positive regulation of protein ubiquitination involved in ubiquitin-dependent protein catabolic process (GO:2000060)	92	11	32	2.8	3.48E-03
Cell division (GO:0051301)	488	61	168	2.8	6.55E-27
G1/S transition of mitotic cell cycle (GO:0000082)	122	15	42	2.8	7.94E-05
Regulation of ubiquitin-protein transferase activity (GO:0051438)	125	16	43	2.8	5.44E-05
Cell cycle checkpoint (GO:0000075)	204	25	70	2.8	1.30E-09
Mitotic cell cycle process (GO:1903047)	647	80	222	2.8	1.84E-36
Regulation of mRNA processing (GO:0050684)	114	14	39	2.8	3.09E-04
Mitotic cell cycle (GO:0000278)	703	87	240	2.8	2.13E-39
Mitotic cell cycle checkpoint (GO:0007093)	153	19	52	2.7	2.27E-06
Regulation of gene silencing (GO:0060968)	106	13	36	2.7	1.20E-03
Regulation of mitotic nuclear division (GO:0007088)	160	20	54	2.7	1.33E-06

Negative regulation of ubiquitin-protein transferase activity (GO:0051444)	86	11	29	2.7	2.14E-02
Regulation of posttranscriptional gene silencing (GO:0060147)	84	10	28	2.7	3.90E-02
Regulation of protein ubiquitination involved in ubiquitin-dependent protein catabolic process (GO:2000058)	108	13	36	2.7	1.86E-03
Regulation of gene silencing by RNA (GO:0060966)	84	10	28	2.7	3.90E-02
DNA integrity checkpoint (GO:0031570)	154	19	51	2.7	7.89E-06
Protein ubiquitination involved in ubiquitin-dependent protein catabolic process (GO:0042787)	140	17	46	2.7	6.45E-05
Mitotic cell cycle phase transition (GO:0044772)	263	33	86	2.6	3.23E-11
Cell cycle phase transition (GO:0044770)	271	34	88	2.6	2.29E-11
DNA damage checkpoint (GO:0000077)	145	18	47	2.6	6.70E-05
RNA splicing, via transesterification reactions (GO:0000375)	288	36	93	2.6	5.16E-12
MRNA splicing, via spliceosome (GO:0000398)	285	35	92	2.6	7.54E-12
RNA splicing, via transesterification reactions with bulged adenosine as nucleophile (GO:0000377)	285	35	92	2.6	7.54E-12
Ribonucleoprotein complex localization (GO:0071166)	128	16	41	2.6	7.93E-04
Negative regulation of mitotic cell cycle phase transition (GO:1901991)	204	25	65	2.6	2.06E-07
Ribonucleoprotein complex export from nucleus (GO:0071426)	126	16	40	2.6	1.41E-03
RNA export from nucleus (GO:0006405)	137	17	43	2.5	6.76E-04
Regulation of nuclear division (GO:0051783)	185	23	58	2.5	4.27E-06
DNA replication (GO:0006260)	215	27	67	2.5	2.58E-07
Negative regulation of translation (GO:0017148)	145	18	45	2.5	4.69E-04
Spindle organization (GO:0007051)	126	16	39	2.5	3.71E-03

[a] In a reference list of 21,042 human genes

[b] In a cluster list of 2,610 genes
[c] Bonferroni-corrected hypergeometric test

Supplemental Table 4 | Enriched Gene Ontology (GO) terms in the magenta cluster in **Fig. 4a**.

		Genes in cluster			
**GO biological process**	**Total genes**	**Expected**	**Observed**	**Fold enrichment**	***p*-value**
Secondary alcohol biosynthetic process (GO:1902653)	42	5	19	3.6	2.12E-02
Regulation of cholesterol biosynthetic process (GO:0045540)	42	5	19	3.6	2.12E-02
Sterol biosynthetic process (GO:0016126)	46	6	20	3.5	2.10E-02
Regulation of cholesterol metabolic process (GO:0090181)	53	7	23	3.5	3.98E-03
Alcohol biosynthetic process (GO:0046165)	84	10	30	2.9	4.72E-03
Secondary alcohol metabolic process (GO:1902652)	116	14	36	2.5	9.66E-03

[a] In a reference list of 21,042 human genes
[b] In a cluster list of 2,612 genes
[c] Bonferroni-corrected hypergeometric test

Supplemental Table 5 | Enriched Gene Ontology (GO) terms in the yellow cluster in **Fig. 4a**.

GO biological process	Total genes[a]	Genes in cluster Expected[b]	Genes in cluster Observed	Fold enrichment	*p*-value[c]
Mitochondrial translational elongation (GO:0070125)	86	14	44	3.1	1.50E-06
Mitochondrial translational termination (GO:0070126)	88	15	45	3.1	9.52E-07
Mitochondrial translation (GO:0032543)	108	18	54	3.0	3.22E-08
Translational termination (GO:0006415)	96	16	46	2.9	4.54E-06
Mitochondrial gene expression (GO:0140053)	124	21	58	2.8	7.03E-08
NADH dehydrogenase complex assembly (GO:0010257)	65	11	30	2.8	8.82E-03
Mitochondrial respiratory chain complex I assembly (GO:0032981)	65	11	30	2.8	8.82E-03
Mitochondrial respiratory chain complex I biogenesis (GO:0097031)	65	11	30	2.8	8.82E-03
Mitochondrial respiratory chain complex assembly (GO:0033108)	95	16	42	2.7	2.28E-04
Translational elongation (GO:0006414)	121	20	53	2.7	5.17E-06
Ribonucleoside monophosphate biosynthetic process (GO:0009156)	77	13	33	2.6	1.28E-02
Respiratory electron transport chain (GO:0022904)	112	19	47	2.5	1.66E-04
RNA modification (GO:0009451)	135	22	56	2.5	1.16E-05
ATP synthesis coupled electron transport (GO:0042773)	92	15	38	2.5	5.20E-03

[a] In a reference list of 21,042 human genes
[b] In a cluster list of 3,480 genes
[c] Bonferroni-corrected hypergeometric test

Supplemental Table 6 | Enriched Gene Ontology (GO) terms in the green cluster in **Fig. 4a**.

GO biological process	Total genes[a]	Genes in cluster Expected[b]	Observed	Fold enrichment	*p*-value[c]
SRP-dependent cotranslational protein targeting to membrane (GO:0006614)	93	7	59	8.2	9.17E-30
Cotranslational protein targeting to membrane (GO:0006613)	99	8	59	7.7	2.36E-28
Protein targeting to ER (GO:0045047)	102	8	59	7.5	1.10E-27
Viral transcription (GO:0019083)	114	9	65	7.4	2.08E-30
Establishment of protein localization to endoplasmic reticulum (GO:0072599)	106	8	59	7.2	7.93E-27
Viral gene expression (GO:0019080)	128	10	69	7.0	5.52E-31
Nuclear-transcribed mRNA catabolic process, nonsense-mediated decay (GO:0000184)	119	9	64	6.9	1.71E-28
Protein localization to endoplasmic reticulum (GO:0070972)	125	10	65	6.7	3.68E-28
Translational initiation (GO:0006413)	143	11	73	6.6	1.89E-31
Protein targeting to membrane (GO:0006612)	133	10	64	6.2	7.55E-26
Cytoplasmic translation (GO:0002181)	46	4	20	5.6	1.22E-05
Nuclear-transcribed mRNA catabolic process (GO:0000956)	199	15	74	4.8	1.86E-23
RRNA processing (GO:0006364)	259	20	93	4.6	4.64E-29
MRNA catabolic process (GO:0006402)	212	16	74	4.5	7.74E-22
Ribosome assembly (GO:0042255)	66	5	23	4.5	4.62E-05
RRNA metabolic process (GO:0016072)	285	22	98	4.4	2.55E-29
Ribosomal large subunit biogenesis (GO:0042273)	70	5	24	4.4	2.96E-05
RNA catabolic process (GO:0006401)	240	19	79	4.3	6.44E-22
Establishment of protein localization to membrane (GO:0090150)	218	17	71	4.2	3.56E-19
Ribosome biogenesis (GO:0042254)	333	26	106	4.1	3.47E-29
Ribosomal small subunit biogenesis	74	6	23	4.0	3.59E-04

(GO:0042274)					
Translation (GO:0006412)	383	30	116	3.9	2.59E-30
Protein targeting (GO:0006605)	277	21	82	3.8	5.81E-20
Peptide biosynthetic process (GO:0043043)	410	32	119	3.8	1.62E-29
NcRNA processing (GO:0034470)	400	31	113	3.7	7.05E-27
Ribonucleoprotein complex biogenesis (GO:0022613)	468	36	127	3.5	5.74E-29
Amide biosynthetic process (GO:0043604)	480	37	126	3.4	2.18E-27
Establishment of protein localization to organelle (GO:0072594)	375	29	95	3.3	7.37E-19
NcRNA metabolic process (GO:0034660)	560	43	140	3.2	1.13E-28
Peptide metabolic process (GO:0006518)	538	42	129	3.1	1.65E-24
Nucleobase-containing compound catabolic process (GO:0034655)	358	28	84	3.0	2.11E-14
Ribonucleoprotein complex assembly (GO:0022618)	208	16	46	2.9	6.16E-06
Protein localization to membrane (GO:0072657)	405	31	89	2.8	1.05E-13
Cellular nitrogen compound catabolic process (GO:0044270)	405	31	88	2.8	3.10E-13
Ribonucleoprotein complex subunit organization (GO:0071826)	221	17	48	2.8	4.71E-06
Heterocycle catabolic process (GO:0046700)	403	31	87	2.8	6.82E-13
Protein localization to organelle (GO:0033365)	607	47	131	2.8	7.40E-21
Cellular amide metabolic process (GO:0043603)	696	54	145	2.7	5.67E-22
Aromatic compound catabolic process (GO:0019439)	418	32	86	2.7	1.52E-11
TRNA metabolic process (GO:0006399)	185	14	38	2.7	1.13E-03
MRNA metabolic process (GO:0016071)	671	52	131	2.5	3.58E-17
Organic cyclic compound catabolic process (GO:1901361)	451	35	88	2.5	1.36E-10
RNA processing (GO:0006396)	887	69	172	2.5	2.77E-23

[a] In a reference list of 21,042 human genes
[b] In a cluster list of 1,630 genes
[c] Bonferroni-corrected hypergeometric test

Supplemental Table 7 | Enriched Gene Ontology (GO) terms in the gray cluster in **Fig. 4a**.

GO biological process	Total genes[a]	Genes in cluster Expected[b]	Genes in cluster Observed	Fold enrichment	*p*-value[c]
Type I interferon signaling pathway (GO:0060337)	65	9	40	4.3	5.05E-10
Cellular response to type I interferon (GO:0071357)	65	9	40	4.3	5.05E-10
Response to type I interferon (GO:0034340)	70	10	42	4.2	2.76E-10
Interferon-gamma-mediated signaling pathway (GO:0060333)	71	10	36	3.6	1.72E-06
Negative regulation of viral genome replication (GO:0045071)	52	7	23	3.1	2.80E-02
Negative regulation of viral process (GO:0048525)	92	13	36	2.8	1.12E-03
Negative regulation of viral life cycle (GO:1903901)	75	11	29	2.7	2.20E-02

[a] In a reference list of 21,042 human genes
[b] In a cluster list of 2,996 genes
[c] Bonferroni-corrected hypergeometric test

Supplemental Table 8 | Enriched Gene Ontology (GO) terms in the yellow cluster in **Fig. 5a**.

GO biological process	Total genes[a]	Genes in cluster Expected[b]	Genes in cluster Observed	Fold enrichment	*p*-value[c]
Mitochondrial translational elongation (GO:0070125)	86	15	56	3.8	8.82E-13
Mitochondrial translational termination (GO:0070126)	88	15	57	3.8	5.93E-13
Translational termination (GO:0006415)	96	16	59	3.6	1.70E-12
Mitochondrial electron transport, NADH to ubiquinone (GO:0006120)	49	8	30	3.6	3.93E-05
NADH dehydrogenase complex assembly (GO:0010257)	65	11	39	3.5	3.77E-07
Mitochondrial respiratory chain complex I assembly (GO:0032981)	65	11	39	3.5	3.77E-07
Mitochondrial respiratory chain complex I biogenesis (GO:0097031)	65	11	39	3.5	3.77E-07
Mitochondrial translation (GO:0032543)	108	18	64	3.5	5.10E-13
ATP synthesis coupled electron transport (GO:0042773)	92	16	53	3.4	6.68E-10
Mitochondrial ATP synthesis coupled electron transport (GO:0042775)	91	15	52	3.4	1.54E-09
Oxidative phosphorylation (GO:0006119)	100	17	57	3.4	1.21E-10
Mitochondrial gene expression (GO:0140053)	124	21	69	3.3	7.09E-13
Inner mitochondrial membrane organization (GO:0007007)	40	7	22	3.2	2.32E-02
Respiratory electron transport chain (GO:0022904)	112	19	61	3.2	1.05E-10
Translational elongation (GO:0006414)	121	21	65	3.2	2.71E-11
Mitochondrial respiratory chain complex assembly (GO:0033108)	95	16	51	3.2	2.47E-08
Electron transport chain (GO:0022900)	115	19	61	3.1	3.22E-10
Cellular protein complex disassembly (GO:0043624)	129	22	66	3.0	1.52E-10
Cellular respiration (GO:0045333)	167	28	85	3.0	3.82E-14

Fatty acid beta-oxidation (GO:0006635)	58	10	29	3.0	4.52E-03
Aerobic respiration (GO:0009060)	60	10	28	2.8	2.55E-02
Ribonucleoside monophosphate biosynthetic process (GO:0009156)	77	13	34	2.6	8.03E-03
Fatty acid catabolic process (GO:0009062)	87	15	38	2.6	2.49E-03
Protein complex disassembly (GO:0043241)	187	32	81	2.6	1.13E-09
Acyl-CoA metabolic process (GO:0006637)	91	15	39	2.5	2.78E-03
Thioester metabolic process (GO:0035383)	91	15	39	2.5	2.78E-03
Energy derivation by oxidation of organic compounds (GO:0015980)	240	41	102	2.5	2.70E-12

[a] In a reference list of 21,042 human genes
[b] In a cluster list of 3,567 genes
[c] Bonferroni-corrected hypergeometric test

Supplemental Table 9 | Enriched Gene Ontology (GO) terms in the yellow cluster in **Fig. 5b**.

		Genes in cluster			
**GO biological process**	**Total genes**[a]	**Expected**[b]	**Observed**	**Fold enrichment**	***p*-value**[c]
Mitochondrial translational termination (GO:0070126)	88	12	30	2.6	4.61E-02
Mitochondrial respiratory chain complex assembly (GO:0033108)	95	13	32	2.5	2.91E-02
Mitochondrial translation (GO:0032543)	108	14	36	2.5	9.37E-03
Translational termination (GO:0006415)	96	13	32	2.5	3.59E-02

[a] In a reference list of 21,042 human genes
[b] In a cluster list of 2,801 genes
[c] Bonferroni-corrected hypergeometric test

Supplemental Table 10 | Enriched Gene Ontology (GO) terms in the purple cluster in **Fig. 5b**.

GO biological process	Total genes[a]	Genes in cluster Expected[b]	Genes in cluster Observed	Fold enrichment	*p*-value[c]
SRP-dependent cotranslational protein targeting to membrane (GO:0006614)	93	20	52	2.6	1.51E-05
Viral transcription (GO:0019083)	114	25	63	2.6	4.99E-07
DNA damage response, signal transduction by p53 class mediator resulting in cell cycle arrest (GO:0006977)	64	14	35	2.5	1.01E-02
Viral gene expression (GO:0019080)	128	28	70	2.5	7.05E-08
Signal transduction involved in mitotic G1 DNA damage checkpoint (GO:0072431)	65	14	35	2.5	1.41E-02
Signal transduction involved in mitotic cell cycle checkpoint (GO:0072413)	65	14	35	2.5	1.41E-02
Signal transduction involved in mitotic DNA integrity checkpoint (GO:1902403)	65	14	35	2.5	1.41E-02
Signal transduction involved in mitotic DNA damage checkpoint (GO:1902402)	65	14	35	2.5	1.41E-02
Intracellular signal transduction involved in G1 DNA damage checkpoint (GO:1902400)	65	14	35	2.5	1.41E-02

[a] In a reference list of 21,042 human genes
[b] In a cluster list of 4,534 genes
[c] Bonferroni-corrected hypergeometric test

Supplementary Table 11 | Conditions for triple-negative breast cancer spheroid culture.

Cell line	Cells per chamber[a]	3D culture medium
BT-549	7500	BT-549 growth medium[b]
HCC38	7500	HCC growth medium[b]
HCC70	10000	HCC growth medium
HCC1143	7500	HCC growth medium
HCC1395	7500	HCC growth medium
HCC1806	5000	HCC growth medium
HCC1937	7500	HCC growth medium
Hs578T	7500	MCF10A assay medium[c]
MCF10A-5E	5000	MCF10A assay medium
MCF10ADCIS.COM	5000	MCF10A assay medium
MDA-MB-231	5000	MCF10A assay medium
MDA-MB-436	7500	MCF10A assay medium
MDA-MB-468	5000	DMEM (Gibco #11965-092) + 10% FBS + Pen/Strep
SUM159PT	5000	SUM159PT growth medium using 2% FBS and Pen/Strep

[a] Using an 8-well chamber slide (Corning #354108)
[b] Growth medium as recommended by ATCC
[c] Assay medium as previously described[1]

REFERENCES

1. Debnath, J., Muthuswamy, S. K. & Brugge, J. S. Morphogenesis and oncogenesis of MCF-10A mammary epithelial acini grown in three-dimensional basement membrane cultures. *Methods* **30**, 256-268 (2003).
2. Huang, L. *et al.* Ductal pancreatic cancer modeling and drug screening using human pluripotent stem cell- and patient-derived tumor organoids. *Nat. Med.* **21**, 1364-1371 (2015).
3. Nguyen-Ngoc, K. V. *et al.* 3D culture assays of murine mammary branching morphogenesis and epithelial invasion. *Methods Mol. Biol.* **1189**, 135-162 (2015).
