# Supplementary material for: Automated brightfield morphometry of 3D organoid populations by OrganoSeg
Source: Sci Rep. 2018 Mar 28;8:5319. doi: 10.1038/s41598-017-18815-8 (PMC5871765; doi:10.1038/s41598-017-18815-8)
Supplement: Supplementary file 2 — Files S1-S3 [file 41598_2017_18815_MOESM2_ESM.doc]

**Supplementary File 1** contains file formats not supported by *Scientific Reports* but can be accessed through the following URL:

<https://virginia.box.com/s/lgp22b7o88t6qpzf1l34vfqhq1kb00or>

**Supplementary File 2** contains file formats not supported by *Scientific Reports* but can be accessed through the following URL:

<https://virginia.box.com/s/nmqfckjl0cfi74k87hxkakg49zy9qftq>

**Supplementary File 3** exceeds the upload limits of NPG but can be accessed through the following URL:

<https://virginia.box.com/s/fopg0y64asyfu2lgbo88r482le9bbuyj>
