# Supplementary material for: Automated brightfield morphometry of 3D organoid populations by OrganoSeg
Source: Sci Rep. 2018 Mar 28;8:5319. doi: 10.1038/s41598-017-18815-8 (PMC5871765; doi:10.1038/s41598-017-18815-8)
Supplement: Supplementary file 6 — SI 1 [file 41598_2017_18815_MOESM6_ESM.zip › BortenMA_OrganoSeg_FileS1/BortenMA_OrganoSeg_Readme.pdf]

OrganoSeg ReadMe

Table of Contents

Installation **3**

Loading New Images **4**

File selection 4

Image resize 4

Segmentation **6**

Out-of-Focus Correction 6

DIC Correction 6

Segmentation Parameters 7

GUI Layout **8**

Spheroid Editing Toolbar **9**

Remove Tool 9

Combine Tool 10

Metric Selection Tool **12**

Pixel Calibration Tool **13**

Save Images **14**

Current Images 14

Image Set 14

Loading Segmented Images **15**

Loading Segmented Images **16**

Currently Known Bugs **17**

Chapter 1: Installation

Installing and running the standalone (.exe/.dmg) implementation

1. Double click the OrganoSeg .exe or .dmg icon.
2. Follow installation instructions. Check “Create Desktop Icon”.
3. Double click the installed OrganoSeg icon.

Installing and running the MATLAB implementation

1. Place the following files into an active MATLAB directory. This can be done by extracting the zipped files into the directory, or by dragging and dropping them.

Files needed:

- adaptivethreshold.m
- imoverlay.m
- OrganoSeg.m
- OrganoSeg.fig
- radialpoly.m
- segmentCurrent
- standaloneCheckBox.m
- standaloneCheckBox.fig
- Zernike_main.m
- Zernikmoment.m

Additional files for MAC:

- xlwrite.m
- dom4j-1.6.1.jar
- poi-3.8-20120326.jar
- poi-ooxml-3.8-20120326.jar
- poi-ooxml-schemas-3.8-20120326.jar
- stax-api-1.0.1.jar
- xmlbeans-2.3.0.jar

2. Ensure the files are all on the MATLAB path.
3. Either load the OrganoSeg.m script and press "Run", or type OrganoSeg into the MATLAB command window.

Chapter 2: Loading New Images

File selection (File → Open → New Image Set or Ctrl+o):
Click the image you wish to load. To load multiple images concurrently, hold shift and select the image that is at the end of the range you would like to upload. To load multiple non-sequential images, hold control and select the additional images. Click open to finalize the upload of the highlighted images.

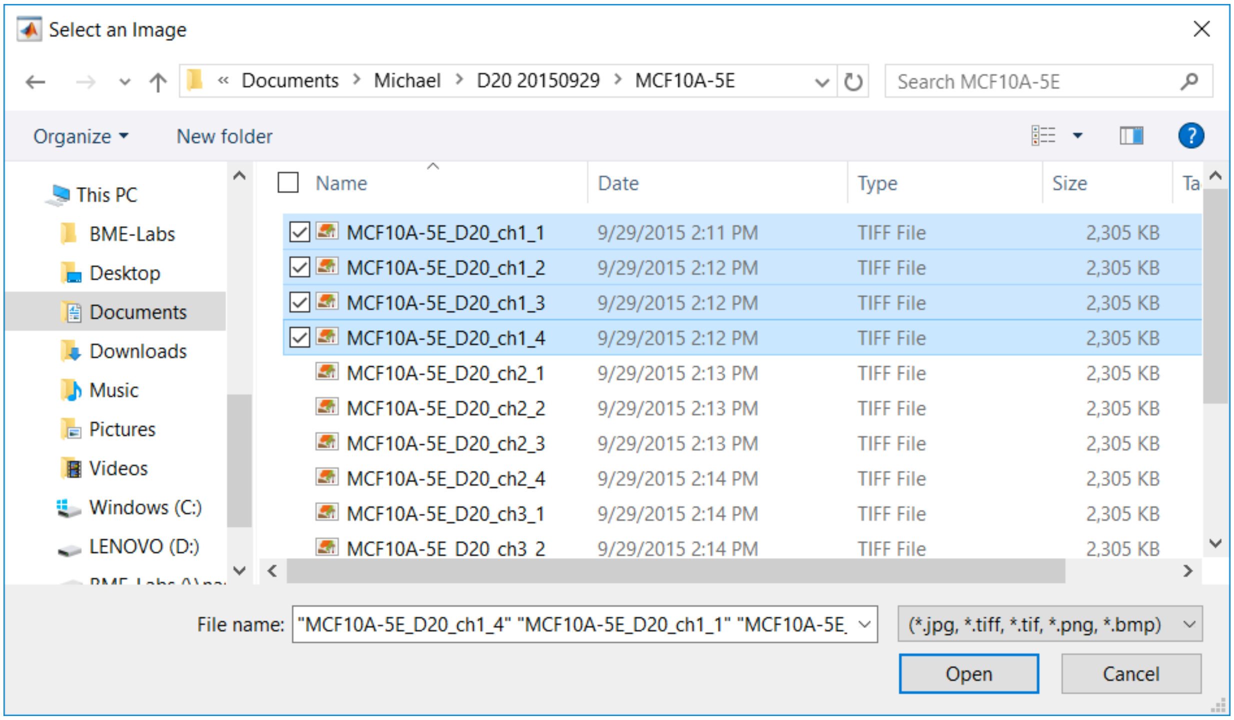

Image resize
The choice to resize large, unbinned images is presented to the user. Image resizing quickens the segmentation by down-sampling large images for adaptive thresholding and then enlarging the segmented boundaries for metric export. Image resizing is recommended if the image height is larger than 768 pixels.

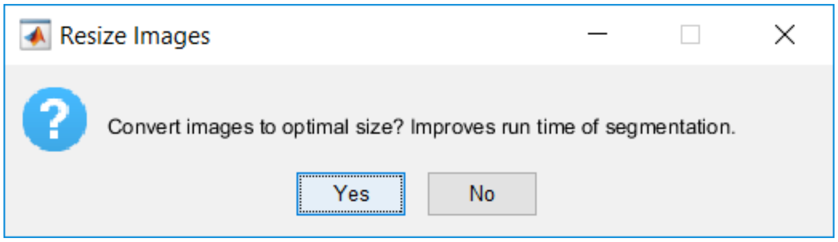

Images will appear in the “Select Image” list. Scrolling through this list will show the selected image in the “Raw Image” panel.

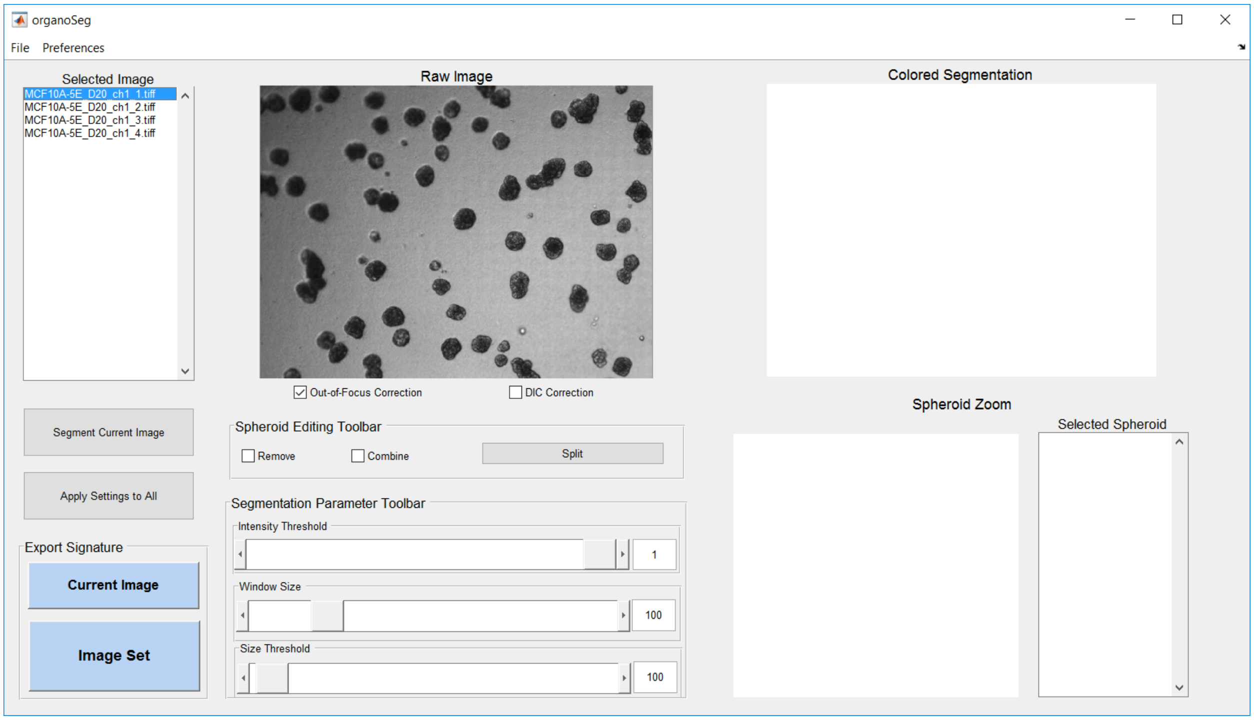

Chapter 3: Segmentation

Selecting or modifying any of the following checkboxes or sliders will automatically segment the selected image:

Out-of-Focus Correction (default: ON):

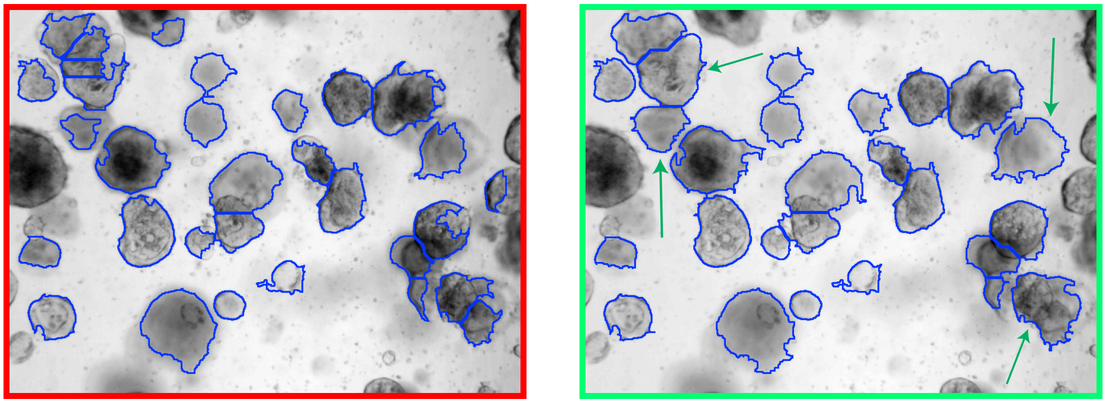

This option performs multi-window thresholding to include blurred content outside of the image plane. The red image displays results with the option OFF; the green outlined image displays results with the option ON. Arrows highlight spheroids that improve upon out-of-focus correction.

DIC Correction (default: OFF):
This correction allows for a tighter identification of the border in DIC images.

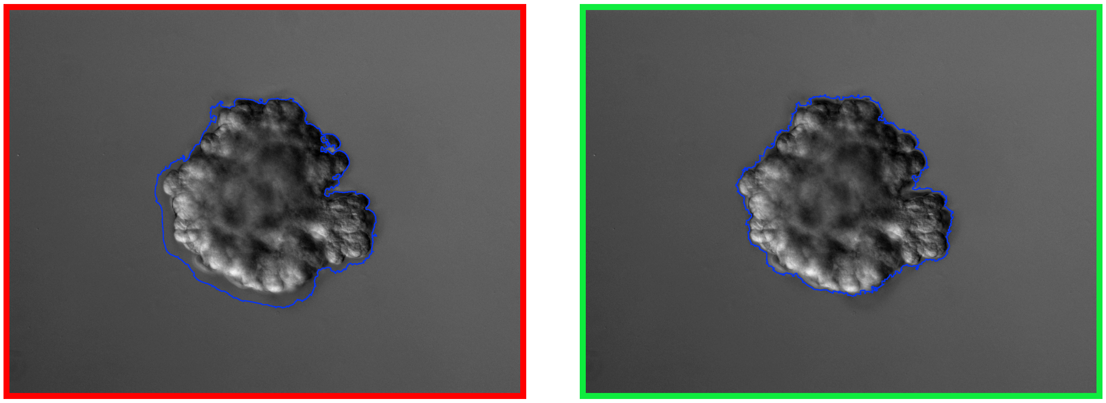

Segmentation parameters

Intensity threshold slider (default: 1 [no modification]):
This slider adjusts a parameter that modulates the Otsu threshold level. Lower threshold level distinguishes finer contrast differences, while higher threshold level requires higher contrast differences to identify an object. Ideal intensity threshold varies from image to image.

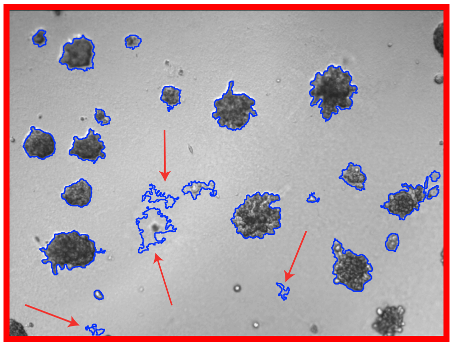

Too Low

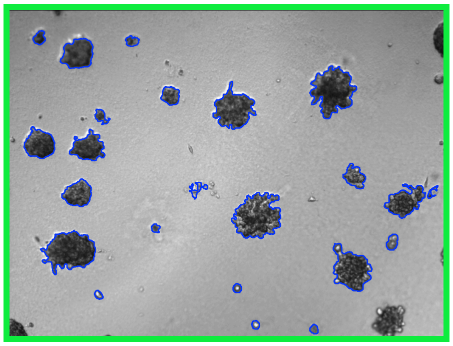

Ideal

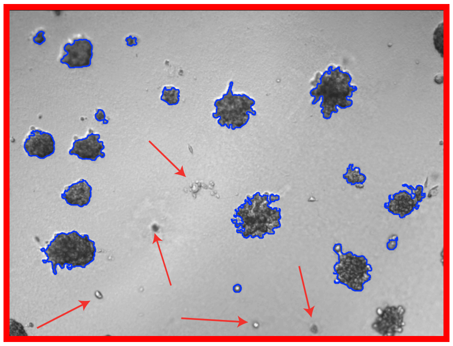

Too High

Window size slider (default: 100 [with out-of-focus correction] or 250 [without out-of-focus correction]):
The window size parameter determines the averaging filter in the adaptive thresholding algorithm. Larger window sizes capture more global detail; smaller window sizes capture more local detail. Ideal window size varies from image to image

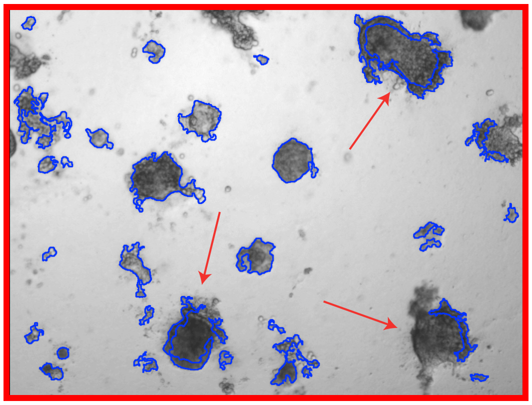

Worse (small)

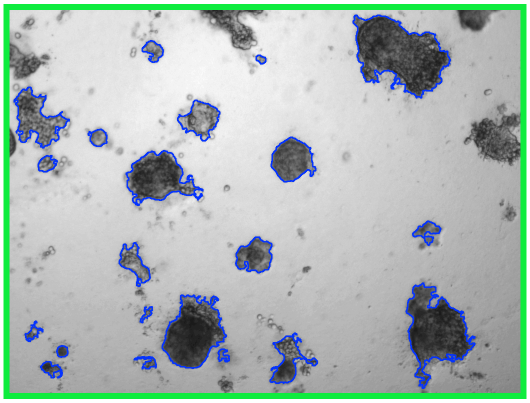

Better (large)

Size threshold (default: 100):
Eliminates any organoids under the specified pixel area value

Using the above features, modify the segmentation until the results are satisfactory for the application. If multiple images contain organoids with similar morphologies, press "Apply Settings to All" to use the current settings for the remainder of the images. After applying settings, additional fine tuning of individual images is possible by clicking on an image in the “Selected Image” list and altering its segmentation parameters.

Chapter 4: GUI layout

After segmenting, a perimeter-overlay will appear in the "Raw Image" panel and a color-labeled image will appear to its right in the "Colored Segmentation" panel. Below the color-labeled image, a zoom-in of the highlighted spheroid will appear. Scrolling through the "Selected Spheroid" list will show a close-up of each spheroid in the image.

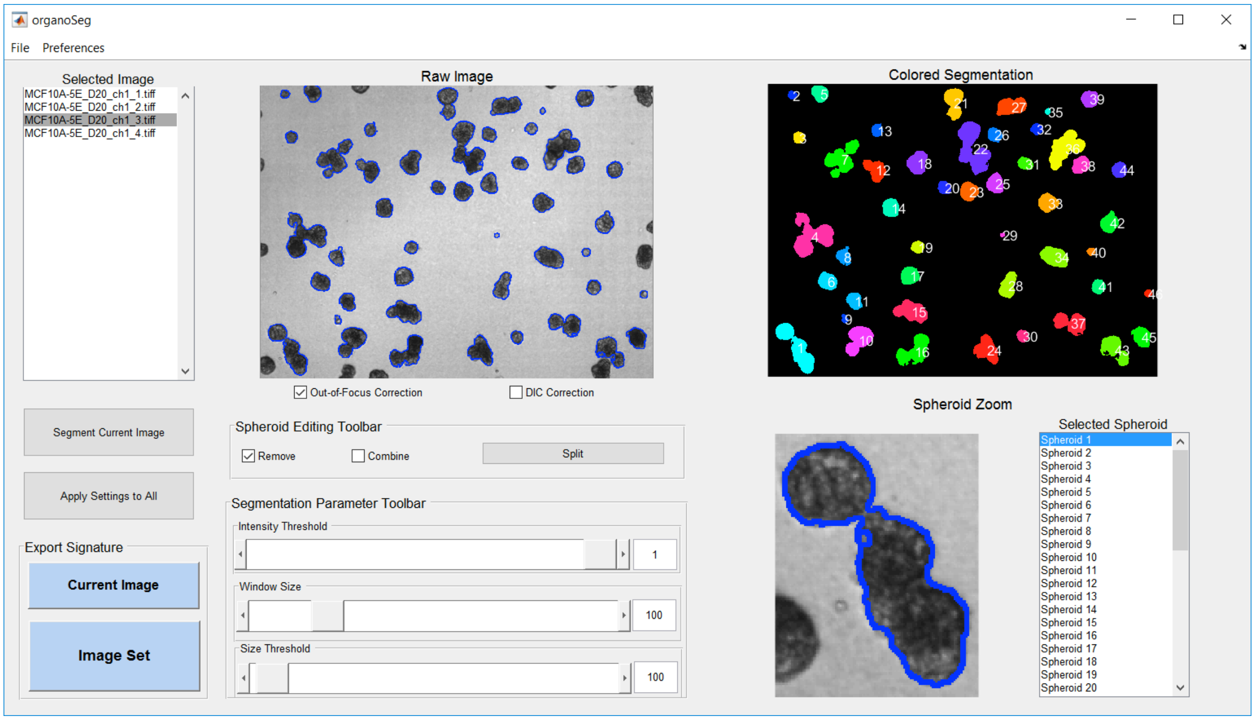

Chapter 5: Spheroid Editing Toolbar

Remove Tool

1. Press the “Remove” toggle button

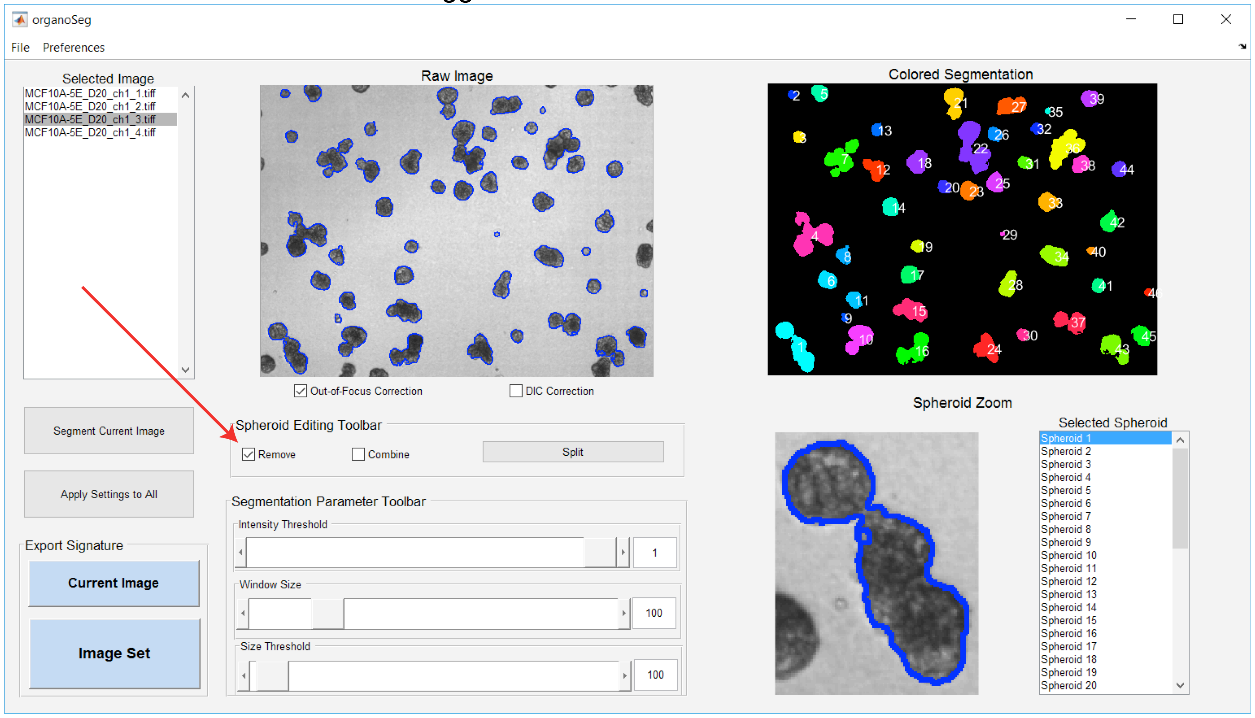

2. Click on a spheroid in the "Raw Image" window that the user wishes to be removed from analysis

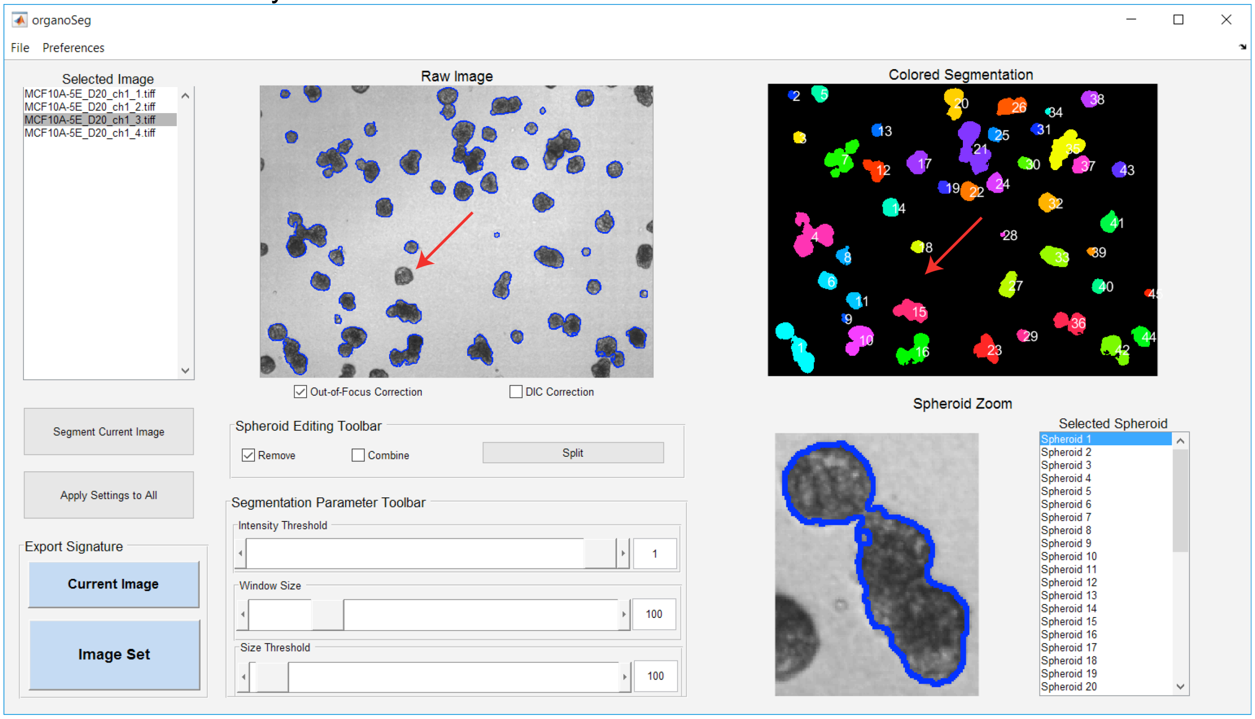

3. Re-click the same spheroid to re-insert its metrics into the data set

Combine Tool

1. Press the check box

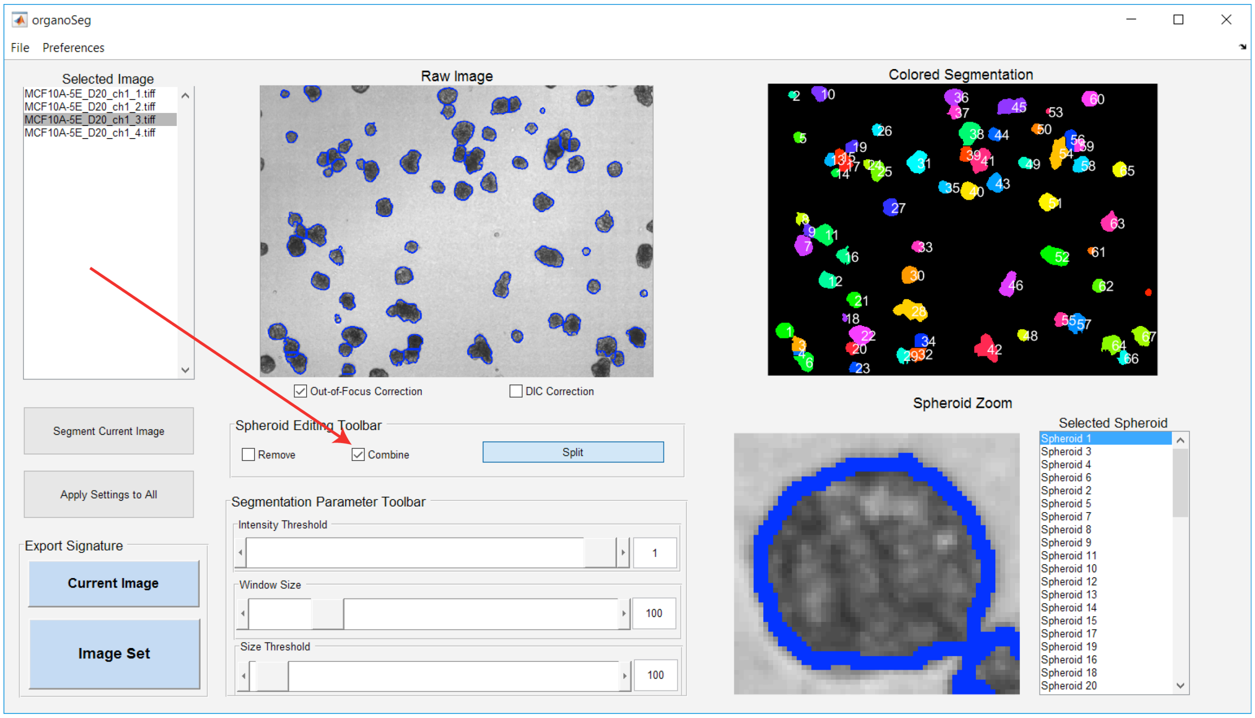

2. Click on the first spheroid the user wishes to combine (red highlight)

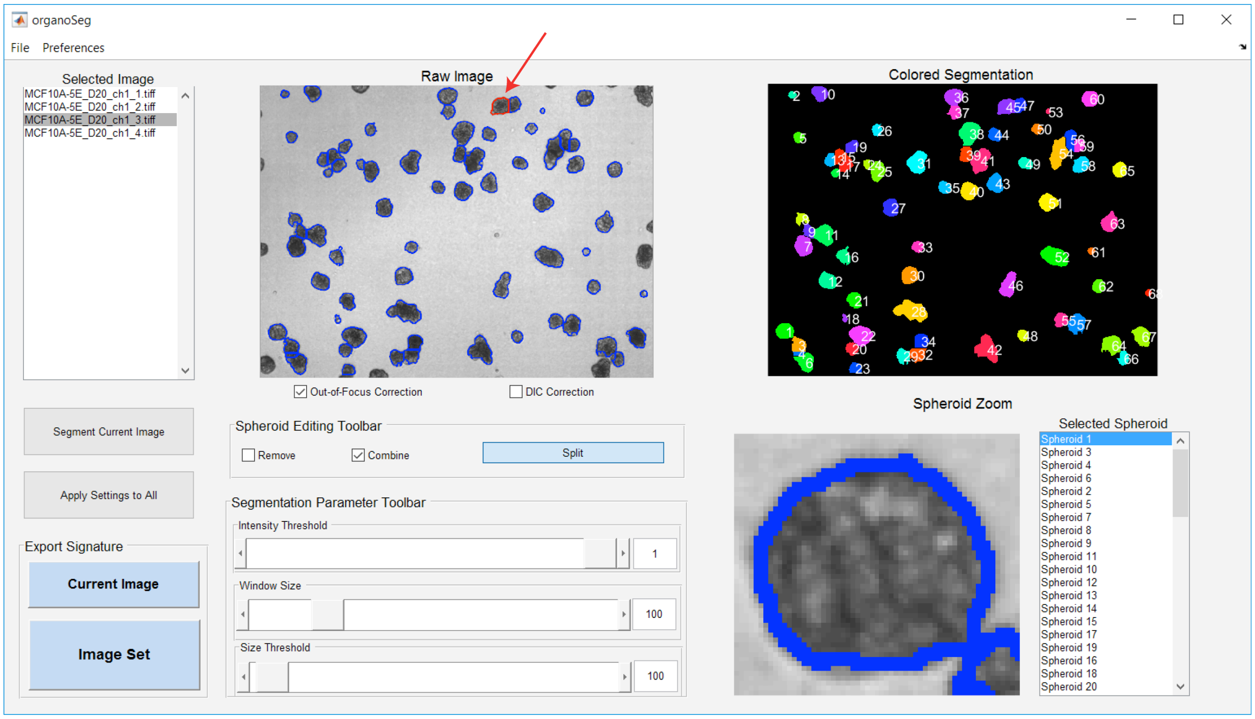

3. Click on the second spheroid the user wishes to combine

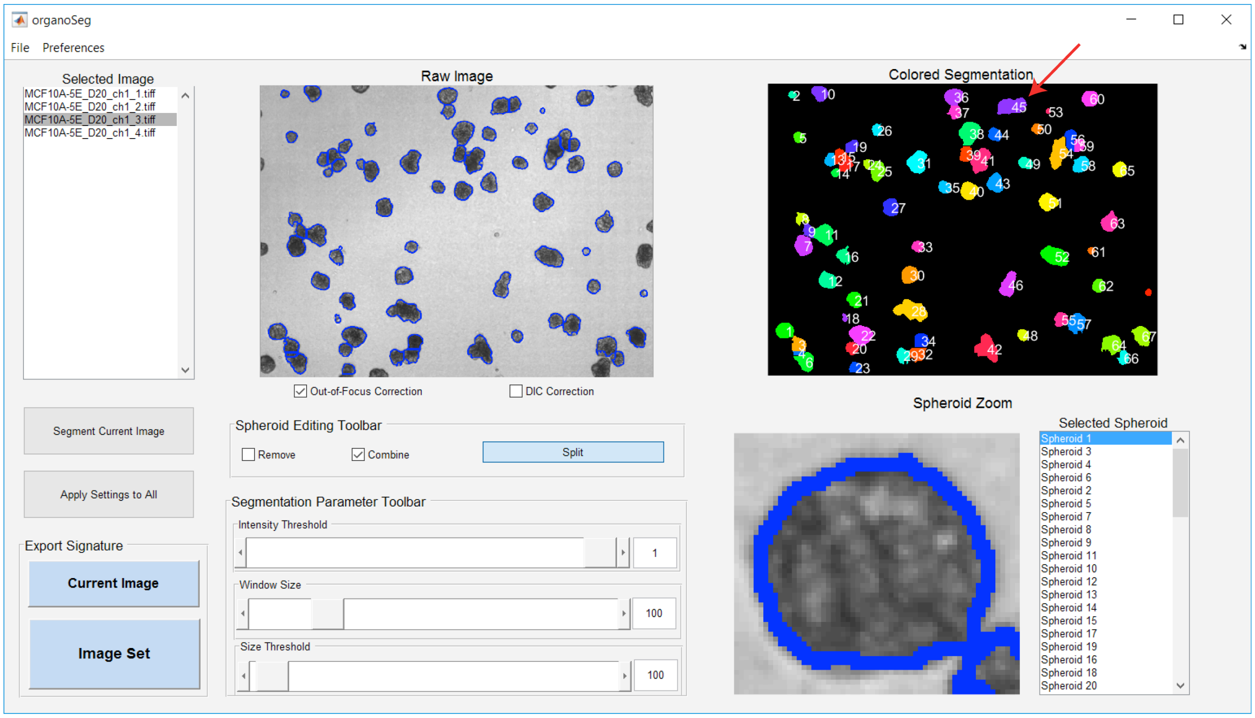

Note 1: The perimeter-overlay does not appear as if these spheroids are combined; however, this is an artifact. The areas are combined as shown in the color-labeled image.

Note 2: Undo not available (see bugs). Must re-segment to separate spheroids, but this will eliminate any other modifications such as removed spheroids.

Chapter 6: Metric Selection Tool (Preferences → Metrics)

The user may select as many of the metrics to export as preferred. The default metrics are: area, perimeter, eccentricity, contrast, correlation, energy, and homogeneity. Click on any blue colored description to view a visual representation in the right panel. The selected metric description will be highlighted in red.

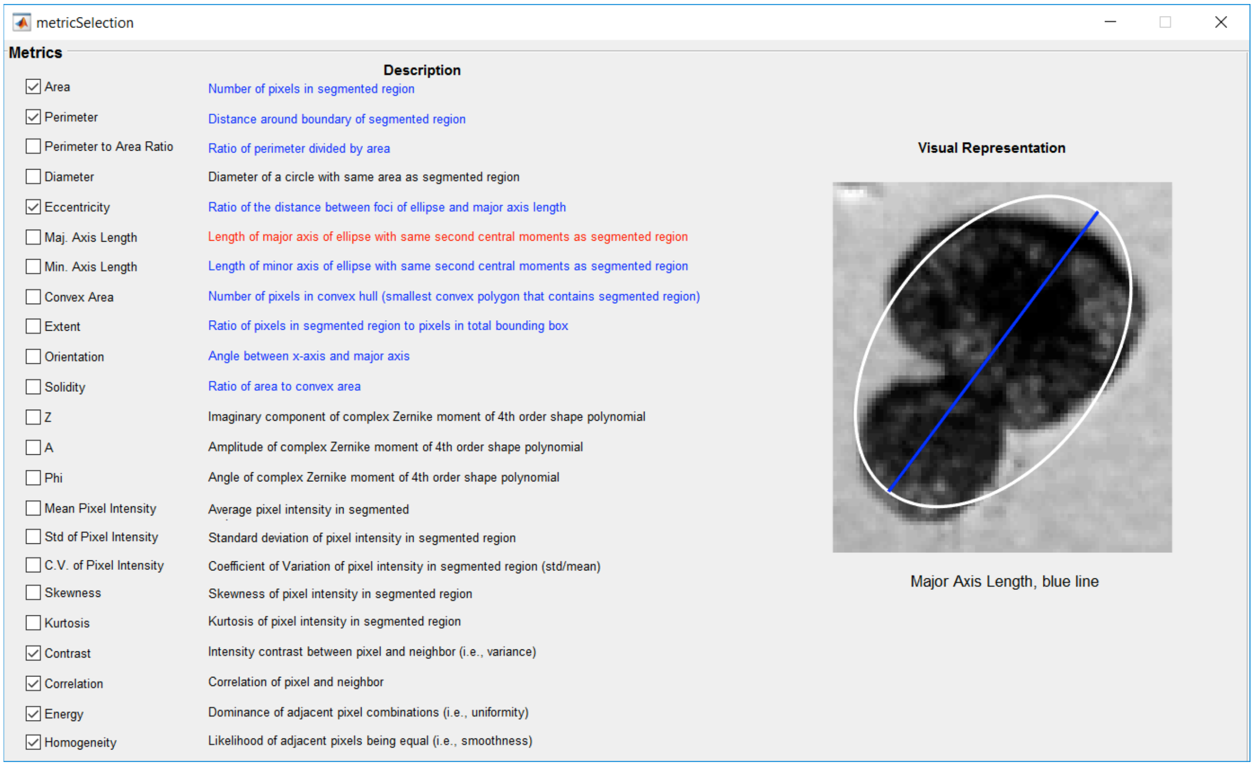

Chapter 7: Pixel Calibration Tool (Preferences → Calibration)

The user may choose a conversion from pixels to microns. The micron-to-pixel ratio will be applied to all exported images in the set.

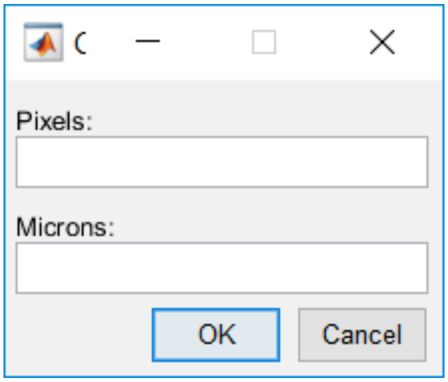

Chapter 8: Save Images (File → Save)

Current Images

Raw-overlay and Colored Images:
Exports the perimeter overlay and color-labeled segmentations as shown in top row of OrganoSeg

Cropped Spheroid:
Exports the zoomed image of the selected spheroid as shown in the bottom right corner of OrganoSeg

Image Set:

Exports the raw-overlay and color-labeled segmentations for all images into the selected folder.

Chapter 9: Export

Under the “Export Signature” panel, select either “Current Image” or “Image Set” to export the respective metric signature. A save window will appear. Choosing *.mat will save metrics, OrganoSeg parameters (Otsu threshold, Max-window size, size-exclusion threshold), images, and processed intermediates, allowing for later modification of the same image set. Choosing *.xls will save only metrics, NOT allowing later modification. To export as *.xls without deleting segmentation data, first save as *.xls, then save as *.mat, **otherwise segmentation data will be lost**.

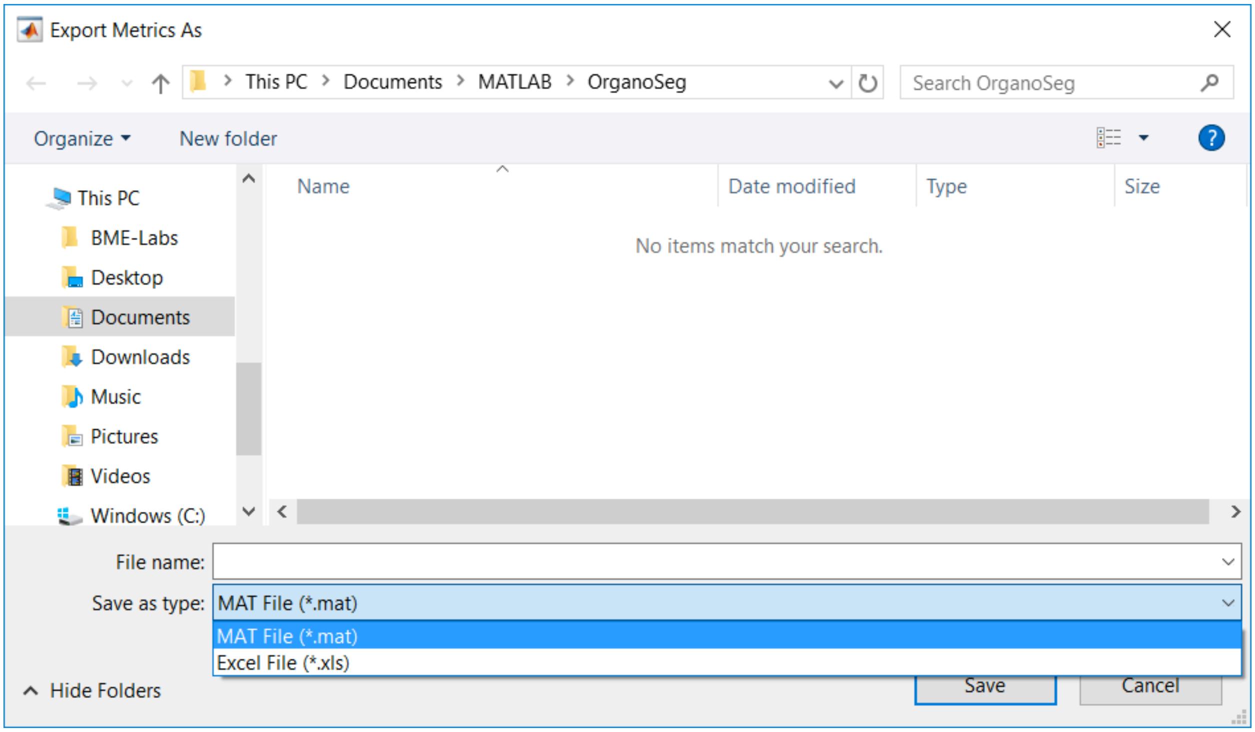

Seven metrics are stored with the default settings: area, perimeter, eccentricity, contrast, correlation, energy, and homogeneity. Metrics may be added or removed with the “Metric Selection Tool” (Preferences → Metrics) as described in Chapter 6.

Chapter 10: Loading Segmented Images

Previously Segmented Images (File → Open → Previously Segmented Images)
The user may select a .mat file of previously segmented images by OrganoSeg. The raw image, colored segmentation image, and spheroid zoom will be displayed for each image in the stack as they were saved.

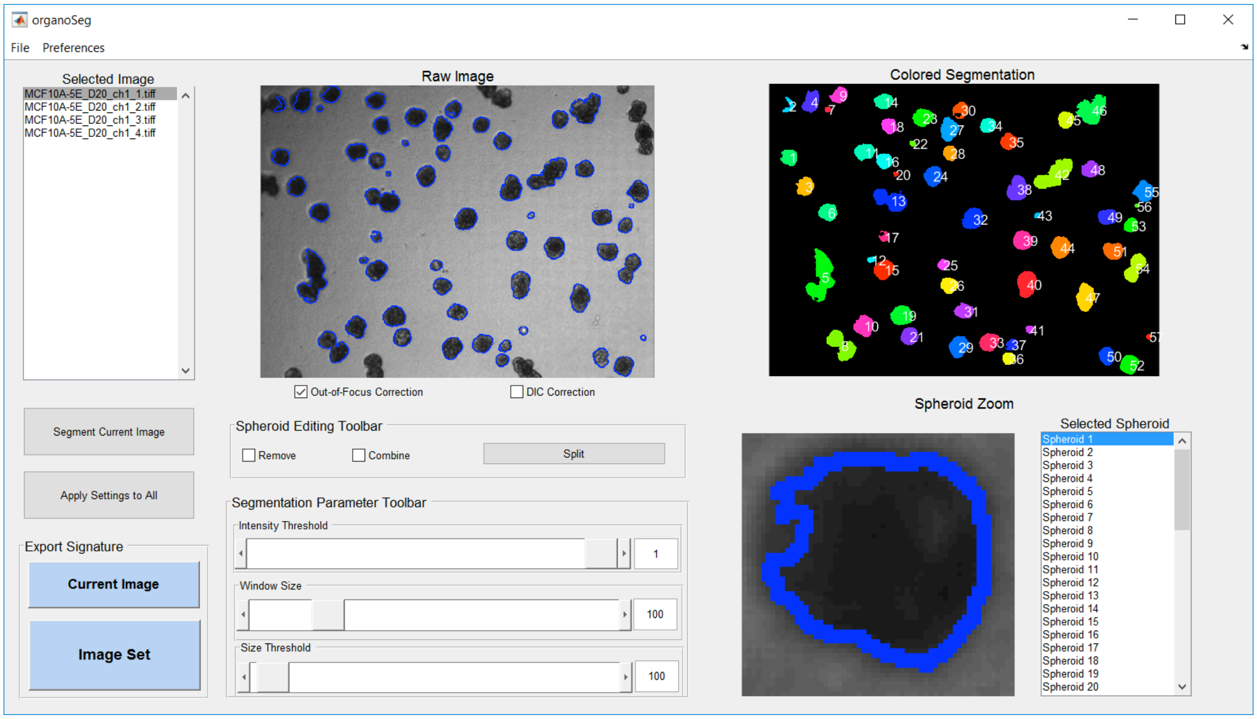

Chapter 11: Currently Known Bugs

- Spheroid re-splitting after using "combine tool" not yet available. If spheroids are combined, the only way to separate them is by re-segmenting, which returns the image to its original state (removed spheroids return, combined spheroids split).
- Spheroids splitting via manual drawing is not yet available
