# Supplementary figures and images for: Automated brightfield morphometry of 3D organoid populations by OrganoSeg
Source: Sci Rep. 2018 Mar 28;8:5319. doi: 10.1038/s41598-017-18815-8 (PMC5871765; doi:10.1038/s41598-017-18815-8)

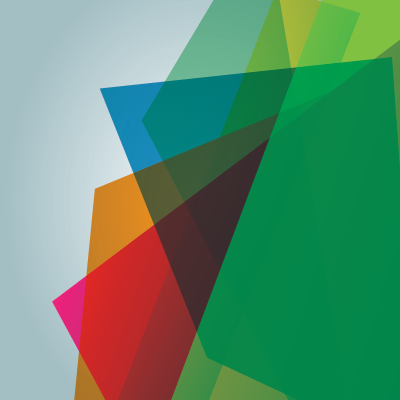

Supplement: Supplementary file 6 — SI 1 [file 41598_2017_18815_MOESM6_ESM.zip › BortenMA_OrganoSeg_FileS1/OrganoSegMACInstaller_web.app/Contents/Resources/splash.png]
